# IL-17 Producing Lymphocytes Cause Dry Eye and Corneal Disease With Aging in RXRα Mutant Mouse

**DOI:** 10.3389/fmed.2022.849990

**Published:** 2022-03-23

**Authors:** Jehan Alam, Ghasem Yazdanpanah, Rinki Ratnapriya, Nicholas Borcherding, Cintia S. de Paiva, DeQuan Li, Rodrigo Guimaraes de Souza, Zhiyuan Yu, Stephen C. Pflugfelder

**Affiliations:** ^1^Department of Ophthalmology, Ocular Surface Center, Baylor College of Medicine, Houston, TX, United States; ^2^Department of Ophthalmology, Baylor College of Medicine, Houston, TX, United States; ^3^Department of Pathology, Washington University School of Medicine, St. Louis, MO, United States; ^4^Department of Ophthalmology, University of São Paulo, São Paulo, Brazil

**Keywords:** gamma delta (gammadelta) T cells, conjunctiva, dry eye, IL-17, retinoic acid, RXR alpha

## Abstract

**Purpose:**

To investigate IL-17 related mechanisms for developing dry eye disease in the Pinkie mouse strain with a loss of function RXRα mutation.

**Methods:**

Measures of dry eye disease were assessed in the cornea and conjunctiva. Expression profiling was performed by single-cell RNA sequencing (scRNA-seq) to compare gene expression in conjunctival immune cells. Conjunctival immune cells were immunophenotyped by flow cytometry and confocal microscopy. The activity of RXRα ligand 9-cis retinoic acid (RA) was evaluated in cultured monocytes and γδ T cells.

**Results:**

Compared to wild type (WT) C57BL/6, Pinkie has increased signs of dry eye disease, including decreased tear volume, corneal barrier disruption, corneal/conjunctival cornification and goblet cell loss, and corneal vascularization, opacification, and ulceration with aging. ScRNA-seq of conjunctival immune cells identified γδ T cells as the predominant IL-17 expressing population in both strains and there is a 4-fold increased percentage of γδ T cells in Pinkie. Compared to WT, IL-17a, and IL-17f significantly increased in Pinkie with conventional T cells and γδ T cells as the major producers. Flow cytometry revealed an increased number of IL-17^+^ γδ T cells in Pinkie. Tear concentration of the IL-17 inducer IL-23 is significantly higher in Pinkie. 9-cis RA treatment suppresses stimulated IL-17 production by γδ T and stimulatory activity of monocyte supernatant on γδ T cell IL-17 production. Compared to WT bone marrow chimeras, Pinkie chimeras have increased IL-17^+^ γδ T cells in the conjunctiva after desiccating stress and anti-IL-17 treatment suppresses dry eye induced corneal MMP-9 production/activity and conjunctival goblet cell loss.

**Conclusion:**

These findings indicate that RXRα suppresses generation of dry eye disease-inducing IL-17 producing lymphocytes s in the conjunctiva and identifies RXRα as a potential therapeutic target in dry eye.

## Introduction

Dry eye is a prevalent disease affecting tens of millions of individuals worldwide (1). Clinical trial results and animal models provide evidence that inflammation contributes to the pathogenesis of ocular surface disease in dry eye (2). The ocular surface is an exposed mucosal tissue that is subjected to desiccating and osmotic stress, as well as microbial danger signals. The conjunctiva has a complement of immune cells that produce factors capable of suppressing sight-threatening inflammation during homeostasis but can respond to pathogen and environmental danger signals. Indeed, ocular surface desiccation has been found to be a potent inflammatory stress that stimulates activation of and production of inflammatory mediators (cytokines, chemokines, and proteases) by the ocular surface epithelial and inflammatory cells (1). This can cause clinical signs of dry eye, such as corneal barrier disruption and conjunctival goblet cell loss (3, 4).

The Lacrimal Functional Unit regulates the production and distribution of tears containing factors that maintain ocular surface epithelial health and suppress ocular surface inflammation (5). One such lacrimal gland secreted factor is vitamin A in the form of retinol that is metabolized to retinoic acid (RA) by the ocular surface epithelium, particularly the conjunctival goblet cells which can deliver it to immune cells located in the underlying stroma (6, 7). Dry eye with corneal and conjunctival epithelial disease develops in systemic vitamin A deficiency; however, the pathogenic mechanisms have not been elucidated. Vitamin A signals through two families of nuclear receptors, the retinoid acid receptor (RAR) and the retinoid X receptor (RXR) that consist as homo- or heterodimers (partners include, RAR, PPAR, vitamin D receptor, and others) (8). RXRα is expressed by a variety of immune cells, including myeloid and lymphoid lineages (9–11) and myeloid cells in the conjunctiva (8). Mice with loss of function mutation in the RXRα nuclear receptor have been reported to develop dry eye (12).

The purpose of this study is to investigate the mechanism for dry eye development in the RXRα loss of function mutant mouse. We found an increased population of IL-17 producing cells consisting of γδ T and conventional T cells in the conjunctival of mice with reduced RXRα signaling that promotes the development of dry eye disease. The RXRα ligand 9-cis RA suppresses the production of IL-17 by γδ T cells and IL-17 inducing cytokines by monocytes.

## Materials and Methods

### Animals

The animal protocol for this study was designed according to the ARVO Statement for the use of Animals in Ophthalmic and Vision Research and was approved by the Institutional Animal Care and Use Committee at Baylor College of Medicine (Protocol AN-2032). Female C57BL/6J (B6) mice and Pepc/BoyJ aged 6–8 weeks were purchased from Jackson Laboratories (Bar Harbor, ME). The RXRα Pinkie mutant strain was purchased from the Mutant Mouse Resource and Research Centers (MRRC, University of California, Davis, Sacramento, CA) for establishing breeder colonies that were expanded in Baylor College of Medicine vivarium and refreshed and genotyped every 8 generations. At the time of the experiments, both B6 and Pinkie strains were housed in the normal vivarium environment.

### Assessment of Corneal Barrier Function and Tear Volume

Corneal epithelial permeability to 70 kDa Oregon-Green-conjugated dextran (OGD; Invitrogen, Eugene, OR) was assessed as previously described (13). Briefly, 1 μL of OGD (50 mg/mL) was instilled onto the ocular surface 1 min before euthanasia; the eye was then rinsed with 2 mL phosphate-buffered saline (PBS) from the temporal and nasal side and photographed with a high-resolution digital camera (Coolsnap HQ2; Photometrics, Tucson, AZ) attached to a stereoscopic zoom microscope (SMZ 1500; Nikon, Melville, NY), under fluorescence excitation at 470 nm. The severity of corneal OGD staining was graded in digital images using NIS Elements (version 3.0; Nikon) within a 2-mm diameter circle placed on the central cornea by 2 masked observers. The mean fluorescence intensity measured by the software inside this central zone was transferred to a database, and the results were averaged within each group. Tear volume was measured with a phenol red impregnated cotton thread as previously described (14).

### Measurement of Goblet Cell Density

Following euthanasia, eyes and ocular adnexa were excised from B6 and Pinkie mice (*n* = 5/group) and the tissues were fixed in 10% formalin followed by paraffin embedding, 5 μm sections were cut with a microtome (Microm HM 340E; Thermofisher Wilmington, DE) and stained with periodic acid Schiff (PAS) reagent. Sections from both eyes in each group were examined and photographed with a microscope (Eclipse E400; Nikon) equipped with a digital camera (DXM1200; Nikon) Using the NIS Elements software; goblet cells were manually counted. To determine the length of the conjunctival goblet cell zone, a line was drawn on the surface of the conjunctiva image from the first to the last PAS^+^ goblet cell. Results are presented as PAS^+^ goblet cells/mm.

### RNA Isolation and Quantitative PCR

Following euthanasia, the cornea/conjunctiva was excised and total RNA was extracted using an RNeasy^®^ Plus Mimi Kit (Cat No. 74134, QIAGEN GmbH, Hilden, Germany) according to manufacturer's instruction. The RNA concentration was measured, and cDNA was synthesized using the Ready-To-Go-You-Prime-First-Strand kit (GE Healthcare). Quantitative real-time PCR was performed with specific probes Murine MGB probes, *Cxcl16* (Mm00801778), *Sprr2a* (Mm00845122_s1), *Sprr2f* (Mm00448855_s1), *Sprr2g* (Mm01326062_m1), *Vegfa* (Mm00437304), *Vegfb* (Mm00442102), *Vegfc* (Mm00437310), *Tnf* (Mm00443260), *Fgf7* (Mm00433291), *Mmp9* (Mm00442991), and hypoxanthine phosphoribosyltransferase (*Hprt1*, Mm00446968). The Hprt-1 gene was used as an endogenous reference for each reaction. The results of real-time PCR were analyzed by the comparative CT method. The CT values of Pinkie were compared to that of B6.

### Tear Washings and Multiplex Immunoassay

Tear-fluid washings were collected from both mouse strains using capillary tubes as previously described (15), and cytokine concentrations in tear samples were assayed using a commercial ProcartaPlex Luminex Assay according to the manufacturer's protocol (Thermofisher). The reactions were detected with streptavidin-phycoerythrin using a Luminex LX200 (Austin, TX, USA) (16). One sample consisted of tear washings from both eyes of 4 mice pooled (8 μL) into a tube containing 8 μL of PBS + 0.1% BSA and stored at −80°C until the assay was performed. Results are presented as the mean ± standard deviation (picograms per milliliter).

### Flow Cytometry and Cell Sorting

Conjunctivae were excised, chopped with scissors into tiny pieces, and incubated with 0.1% type IV Collagenase for 1 h to yield single-cell suspensions. Samples were incubated with anti-CD16/32 (2.4G2, Catalog no. 553141, BD Pharmingen™, San Diego, CA), for 5 min at room temperature and subsequently stained with anti-CD45 (clone 30-F11, Catalog no. 103138, BioLegend) and with an infra-red fluorescent viability dye (Life Technologies, Grand Island, NY). The gating strategy was as follows: lymphocytes were identified by forward -scatter area (FSC-A) and side scatter area (SSC-A) gates, followed by two singlets gates (FSC-A vs. FSC-W and SSC-A vs. SSC-W) followed by live/dead identification using the infra-red fluorescent viability dye. The CD45+ cells were sorted using the Aria-II cell sorter at the Baylor College of Medicine cytometry and cell sorting core.

Antibodies for phenotyping IL-17^+^ cells in the conjunctiva included: anti-CD45 (clone 30-F11, Catalog no. 103138, BioLegend), Alexa Fluor® 488 anti-mouse CD45.1 (Clone A20, catalog #110718, BioLegend Way San Diego, CA), Brilliant Violet 510™ anti-mouse CD45.2 (Clone 104, catalog # 109838, BioLegend Way San Diego, CA), PerCP/Cyanine5.5 anti-mouse CD3ε (Clone 500A2, catalog # 152312, BioLegend Way San Diego, CA), PE Anti-Mouse γδ T-Cell Receptor (Clone GL3, catalog #553178, BD Pharmingen™, San Diego, CA), Alexa Fluor® 647 anti-mouse IL-17A (Clone TC11-18H10, catalog# 560184, BD Pharmingen™, San Diego, CA). A violet live/dead fixable dye (Life Technologies) was used to exclude dead cells. A Canto II flow cytometer (BD Biosciences) and FlowJo 7.6.5 software (TreeStar, Ashland, OR, USA) were used for analysis.

### Library Preparation

Single-cell gene expression libraries were prepared using the Chromium Single Cell Gene Expression 3v3.1 kit (10× Genomics) at the Single Cell Genomics Core at Baylor College of Medicine. In brief, single cells, reverse transcription (RT) reagents, Gel Beads containing barcoded oligonucleotides, and oil were loaded on a Chromium controller (10× Genomics) to generate single-cell Gel Beads-In-Emulsions (GEMs) where full-length cDNA was synthesized and barcoded for each single cell. Subsequently the GEMs are broken and cDNA from every single cell is pooled. Following cleanup using Dynabeads MyOne Silane Beads (Thermofisher, Waltham, MA), cDNA is amplified by PCR. The amplified product is fragmented to optimal size before end-repair, A-tailing, and adaptor ligation. The final library was generated by amplification.

### Sequencing of 10X GEM 3′v3.1 Single Sell Libraries

The BCM Genomic and RNA Profiling (GARP) Core initially conducted sample quality checks using the NanoDrop spectrophotometer and Agilent Bioanalyzer 2100. To quantitate the adapter-ligated library and confirm successful P5 and P7 adapter incorporations, the Applied Biosystems ViiA7 Real-Time PCR System and a KAPA Illumina/Universal Library Quantification Kit (p/n KK4824) was used. The GARP core sequenced the libraries on the NovaSeq 6000 Sequencing System using the S2 v1.0 Flowcell as follows. Cluster Generation by Exclusion Amplification (ExAMP): Using the concentration from the ViiA7 TM qPCR machine above, 150 pM of the equimolar pooled library was loaded onto one lane of the NovaSeq S2 v1.0 flowcell (Illumina p/n 20012860) following the XP Workflow protocol (Illumina kit p/n 20021664) and amplified by exclusion amplification onto a nanowell-designed, patterned flowcell using the Illumina NovaSeq 6000 sequencing instrument. PhiX Control v3 adapter-ligated library (Illumina p/n FC-110-3001) was spiked-in at 1% by weight to ensure balanced diversity and to monitor clustering and sequencing performance. The libraries were sequenced according to the 10X Genomics protocol, 28 cycles for Reads 1, 10 cycles each for the i7 and i5 reads, and 90 cycles for Read 2. An average of 251 million read pairs per sample was sequenced. FastQ file generation was executed using bcl2fastq and QC reports were generated using CellRanger v5.0.1 by the BCM Multiomics Core.

### Bioinformatic Analysis of ScRNA-Seq Data

Raw sequence reads in the FASTQ format were aligned to the mouse reference genome using Cell Ranger Count v6.0.1 pipeline (https://cloud.10xgenomics.com) with the default settings for alignment, barcode assignment, and UMI counting of the raw sequencing data with genome reference Mouse (mm10) 2020-A. The resulting gene expression matrix was subjected to preprocessing following the guideline provided by Seurat v4.1.0. Briefly, single cells with fewer than 200 genes were filtered to remove empty droplets. We also removed the genes that were expressed in <3 cells in our data. Next, we employ a global-scaling normalization method using the Seurat function “LogNormalize” that normalizes the feature expression.

### Clustering, Visualization and Cell Annotation

First, we used the “FindVariableFeatures” function to identify a set of 2,000 genes that are highly variable in the two data sets, and the “FindIntegrationAnchors” and “IntegrateData” functions combined the two data sets for downstream analysis such as dimensionality reduction and clustering. We then performed Principal Components Analysis (PCA) to construct a linear dimensionality reduction of the dataset and identified the 19 PCs that contain most of the complexity of the dataset. The cells were clustered in a graph-based approach within PCA space, and then non-linear dimensionality reductions were applied using UMAP for further visualization purposes. We then used a set of canonical cell type markers to assign annotation to each cluster using the Cluster Identity Predictor (CIPR) web-based tool (https://aekiz.shinyapps.io/CIPR/). Finally, differential expression was performed using the “FindAllMarkers” function in Seurat to find cluster-specific marker genes.

### Monocyte Purification and *in vitro* Stimulation

Bone marrow isolated cells were cultured (2 × 10 ^∧^ 7 cells/100 mm tissue culture dish) in 10 ml of complete medium [RPMI 1640 supplemented with 10% heat inactivated fetal calf serum, 50 μg/ml gentamycin and 1.25 μg/ml amphotericin B (all from Gibco Thermofisher)] containing 20 ng/ml GM-CSF (Peprotech, Inc. USA). Monocytes were purified after 3 days of culture using the monocyte isolation kit, according to the manufacturer's instruction (BM, Miltenyi Biotec, Bergisch Gladbach, Germany). 5 × 10 ^∧^ 5 monocytes plated in a 48 well-plate were preincubated with 100 nM 9-cisRA for 1 h followed by stimulation with 0.5 μg/ml LPS for 4 h for RNA or overnight for cytokines. The total RNA was extracted using an RNeasy^®^ Plus Mimi Kit (Cat No. 74134, QIAGEN GmbH, Hilden, Germany) according to manufacturer's instructions. The RNA and collected supernatants were stored at −80^0^C until further use.

### γδT Cell Isolation and *in vitro* Experiments

Pooled γ/δ T cells from the spleens of 8–10 week old B6 and Pinkie mice were isolated using the TCR γ/δ T cells Isolation Kit according to the manufacturer's instruction (Miltenyi Biotec, Bergisch Gladbach, Germany). To determine the effect of 9CisRA and monocytes conditional media on IL17 cytokine production, we stimulated the purified γδ T cells with anti-CD3/CD28 Dynabeads (Catalog #11452D, Life Technologies AS, Norway) alone or in combination with IL-23 (10 ng/ml, eBioscience), 9-cisRA (100 nM), or monocyte conditioned media for 96 h for cytokine measurement.

### IL-17 ELISA

Mouse IL17 (both heterodimers, A and F) was measured in purified γδT cultured cell supernatant after 96 h incubation using a mouse IL-17 DuoSet Enzyme-linked immunosorbent assay (ELISA) (Catalog no. DY5390-05, R&D Systems, Minneapolis, USA).

### NanoString NCounter Gene Expression Analysis

This was performed by the Genomic and RNA Profiling Core at Baylor College of Medicine using the NanoString Technologies nCounter Gene Expression Mouse Myeloid Innate Immunity V2 Panel codeset (NS_MM_Myeloid_V2.0) containing 770 unique pairs of 35–50 bp reporter probes and biotin-labeled capture probes, including internal reference controls (NanoString, Seattle, WA) as previously described (17). Data was analyzed by ROSALIND^®^ (https://rosalind.bio/), with a HyperScale architecture developed by ROSALIND, Inc. (San Diego, CA).

### Bulk RNA Seq and Data Analysis

Conjunctival epithelium was excised from B6 and Pinkie strains and total RNA was extracted using a QIAGEN RNeasy Plus Micro RNA isolation kit (Qiagen) according to the manufacturer's instructions. The concentration and purity of RNA was assessed using a NanoDrop 1,000 (ThermoFisher Scientific, Waltham, MA). RNA-Seq was performed by the Beijing Genomics Institute (BGI) using the BGISEQ500RS to generate 100-bp paired-end reads. The sequencing reads were cleaned by removing reads containing adapter or poly-N sequences, and reads of low quality using SOAPnuke (version 1.5.2, parameters: -l 15 -q 0.2 -n 0.05). and the expression levels of the resulting genes and transcripts were determined using RSEM (version 2.2.5, default parameters). A total of 19,511 genes were obtained as raw data. Detection of DEGs (differentially expressed genes) was performed with DEseq2 (Parameters: Fold Change >2.00 and adjusted *P* < 0.05). Genes were passed through the Benjamini-Hochberg procedure to obtain the critical value for false discovery and a total of 1,375 genes passed with a *P* >0.0006. The selected genes in the IL-17 signaling pathway were clustered in a heat map using GraphPad Prism 9.0 software (San Diego, CA, USA).

### ATAC Seq

Cultured bone marrow-derived monocytes were harvested and frozen in culture media containing FBS and 5% DMSO. Cryopreserved cells were sent to Active Motif (Carlsbad, CA) to perform the ATAC-seq assay. The cells were then thawed in a 37°C water bath, pelleted, washed with cold PBS, and tagmented as previously described (18), with some modifications (19). Briefly, cell pellets were resuspended in lysis buffer, pelleted, and tagmented using the enzyme and buffer provided in the Nextera Library Prep Kit (Illumina, San Diego, CA). Tagmented DNA was then purified using the MinElute PCR purification kit (Qiagen, Germantown, MD), amplified with 10 cycles of PCR, and purified using Agencourt AMPure SPRI beads (Beckman Coulter, Brea, CA). The resulting material was quantified using the KAPA Library Quantification Kit for Illumina platforms (KAPA Biosystems, St Louis, MO), and sequenced with PE42 sequencing on the NextSeq 500 sequencer (Illumina).

Analysis of ATAC-seq data was similar to the analysis of ChIP-Seq data. Reads were aligned using the BWA algorithm (mem mode; default settings). Duplicate reads were removed, only reads mapping as matched pairs and only uniquely mapped reads (mapping quality ≧ 1) were included for further analysis. Alignments were extended *in silico* at their 3′-ends to a length of 200 bp and assigned to 32-nt bins along the genome. The resulting histograms (genomic “signal maps”) were stored in bigWig files. Peaks (accessible regions) were identified using the MACS (version 2.1.0) at a cutoff of *p*-value 1e-7, without control file, and with the–nomodel option. Peaks that were on the ENCODE blacklist of known false ChIP-Seq peaks were removed. Signal maps and peak locations were used as input data to Active Motifs proprietary analysis program, which creates excel tables containing detailed information on sample comparison, peak metrics, peak locations and gene annotations. For differential analysis, reads were counted in all merged peak regions (using Subread), and the replicates for each condition were compared using DESeq2. The position and frequency of motif sequences in each peak region were identified with the search tool HOMER or known sequences in databases (20).

### Qiagen Gene Pathway Analysis

Briefly, differentially expressed genes from single-cell RNA seq data were first uploaded into Qiagen's Ingenuity Pathway Analysis (IPA) system for core analysis. Analysis was performed with experimental false discovery rate of >0.05. Comparison analysis tool were used to identify the most relevant canonical pathways enriched in Pinkie and presented as a heatmap. IL17 signaling pathway was adopted from IPA with some modification.

### Creation of Bone Marrow Chimeras

CD45.2^+^ bone marrow chimeras using bone marrow cells obtained from 12 to 16 week B6 and Pinkie strains were created in 6–8 week old CD45.1^+^ Pepc/BoyJ strain as previously reported (17, 21). Ten days after bone marrow reconstitution, mice were subjected to 5 days of desiccating stress (DS5) and T cell populations in the conjunctiva were analyzed by flow cytometry.

### Desiccating Stress and IL-17 Neutralization

As previously described (17), DS was induced by inhibiting tear secretion with scopolamine hydrobromide (Greenpark, Houston) in drinking water (0.5 mg/mL) and housing in a cage with a perforated plastic screen on one side to allow airflow from a fan placed 6 inches in front of it for 16 h/day for 5 consecutive days. Room humidity was maintained at 20–30%. Control mice were maintained in a non-stressed (NS) environment at 50–75% relative humidity without exposure to an air draft.

Mice were injected intraperitoneally every 2 days with 100 μg/mouse of anti-IL-17A (Clone 17F3; BioXcell) or mouse IgG1 isotype control (Clone MOPC-21; BioXcell) starting on day −2 for the duration of DS. After 5 days of DS, mice were euthanized and immune cells were harvested from the conjunctiva for flow cytometry (*n* = 11), eyes were embedded in paraffin for sectioning (*n* = 5) or in optimum cutting temperature (OCT) compound (Thermofisher) for cryosectioning (*n* = 3), or corneas were prepared for whole-mount immunostaining (*n* = 3).

### Immunofluorescence Staining and Confocal Microscopy

The conjunctival and corneal tissue samples were dissected from female C57BL/6J mice (age 16 weeks) and fixed in 100% methanol for 20 min at −20°C followed by washing with Hanks' buffered saline solution (HBSS) for 3 × 5 min with gentle shaking at room temperature (RT). Tissues were permeabilized with 0.4% Triton X-100 in HBSS for 30 min at RT and gentle shaking. Twenty percentage goat serum (Sigma, USA) diluted in HBSS was used for 1 h blocking at RT. Subsequently, the conjunctival tissue samples were incubated with primary antibodies (Supplementary Table 1) diluted in 5% goat serum in HBSS at the mentioned concentrations overnight at 4°C with gentle shaking at dark. The samples were then washed with 0.4% Triton X-100 for 3 × 6 min at RT with gentle shaking, followed by incubation with secondary antibodies (Supplementary Table 1) diluted in 5% goat serum/HBSS for 1 hour at RT with gentle shaking and light protection. The samples were then washed for 3 × 10 min with 0.4% Triton X-100 in HBSS and Hoechst (1:500 in HBSS) was added for nuclei staining (30 min at RT and dark with gentle shaking). The samples were washed 3 × 5 min with HBSS, mounted on slides, and flattened with coverslips. Immunofluorescence staining in whole-mount conjunctival tissue samples was visualized using laser scanning Nikon confocal microscope (Nikon A1 RMP, Nikon, Melville, NY, USA) and 0.5 μm Z-step. The captured images were processed using NIS Elements Advanced Research (AR) software version 4.20 (Nikon).

### *In situ* Zymography

*In situ* zymography was performed to localize the gelatinase activity in corneal cryosections using a previously reported method (22). Sections were thawed and incubated overnight with reaction buffer, 0.05 M Tris HCl, 0.15 M NaCl, 5 mM CaCl_2_, and 0.2 mM NaN_3_, pH 7.6, containing 40 mg/ml FITC-labeled DQ gelatin, which was available in a gelatinase/collagenase assay kit (EnzChek, Thermofisher). As a negative control, 50 mM 1,10-phenanthroline, a metalloproteinase inhibitor, was added to the reaction buffer before applying the FITC- labeled DQ gelatin to frozen sections. Proteolysis of the FITC-labeled DQ gelatin substrate yields cleaved gelatin- FITC peptides that are fluorescent at sites of net gelatinolytic activity. After incubation, the sections were washed three times with PBS for 5 min, counterstained with Hoechst 33,342 dye and a coverslip was applied. Areas of gelatinolytic activity of MMPs were viewed and imaged.

### Statistical Analysis

Based on normality, parametric student T or non-parametric Mann–Whitney *U*-tests were performed for statistical comparisons with an alpha of 0.05 using GraphPad Prism 9.0 software.

## Results

### Keratoconjunctivitis Develops in the *Pinkie* Strain With Reduced Rxrα Signaling

Du et al. reported the Pinkie mouse strain, with a loss of function RXRα mutation (I273N) (12) that alters ligand binding and heterodimerization resulting in a 90% decrease in ligand-inducible transactivation, develops signs of dry eye with aging, but the study did not evaluate the ocular surface disease and immunopathology (12). Corneal epithelial barrier disruption, loss of conjunctival goblet cells and increased expression of cornified envelope precursors by the surface epithelium are well-characterized pathological features of dry eye disease (23, 24).

**Figure 1:**
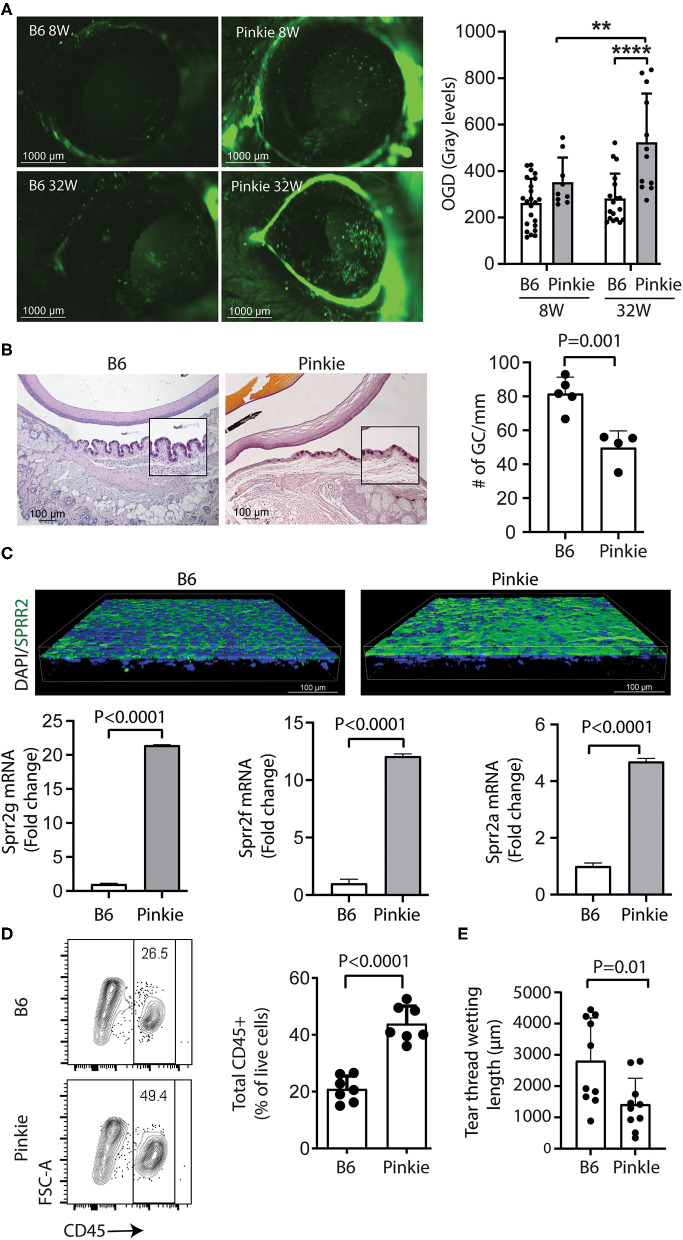
The Pinkie strain with reduced Rxrα signaling develops dry eye disease. **(A)** Dry eye phenotype in Pinkie strain. Representative Oregon Green dextran (OGD) staining of corneas from 8- to 32-week-old C57BL/6 (B6) and Pinkie strains (left) and graph showing mean gray level fluorescence (*n* = 9–22/group). Mean ± SD, **P < 0.01; ****P < 0.0001; **(B)** Representative images of PAS staining of conjunctival goblet cells in paraffin sections prepared from B6 and Pinkie strains (left) with bar graph of mean goblet cell density (right), *n* = 5/group. **(C)** Representative SPPR2 immunostaining of whole-mount conjunctivas (age 8–10 weeks) stained with SPRR2 polyclonal antibody that recognizes multiple isoforms and nuclei stained with Hoechst 33343 dye. Images are captured by confocal microscopy; relative fold expression of *Sprr2g, Sprr2f*, and *Sprr2a* genes in Pinkie conjunctiva (below), *n* = 5/group. **(D)** Flow cytometry scatter plot showing increased percentage of CD45^+^ cells in the Pinkie conjunctiva (left) and bar graph comparing CD45^+^ cells in conjunctiva obtained from B6 and Pinkie (*n* = 7). **(E)** Decreased tear volume in Pinkie (age 8–10 week) measured by phenol red cotton thread as compared to B6.

Corneal staining after topically applied 70 kDa Oregon Green Dextran (OGD) increases with corneal barrier disruption in dry eye. There is no statistical difference in corneal OGD permeability between younger (8W old) Pinkie and wild type (WT) C57BL/6 (B6) but OGD staining is significantly increased in 32-week-old *Pinkie* (Figure 1A). Reduction in conjunctival goblet cell number is another marker of dry eye. Pinkie has a significantly reduced number of PAS-positive conjunctival goblet cells at 8 weeks of age, compared to the WT strain (Figure 1B). Increased immunoreactivity to the cornified envelope precursor SPRR2 in the conjunctival epithelium (Figure 1C, left) in Pinkie is accompanied by increased expression of several *Sprr* isoform genes in the conjunctiva [*Sprr2g* (>20 fold), *Sprr2f* (>10 fold) and *Sprr2a* (>4 fold) compared to B6 (Figure 1C, right and below). Pinkie at 8–10 weeks of age has a significantly increased number of CD45^+^ immune cells in the conjunctiva by flow cytometry (Figure 1D) and decreased tear volume (Figure 1E). These findings indicate Pinkie has dry eye-associated pathological changes in the corneal and conjunctival epithelia and the corneal epithelial disease worsens with age.

### Pinkie Has Increased IL-17 Producing Lymphocytes in the Conjunctiva

**Figure 2:**
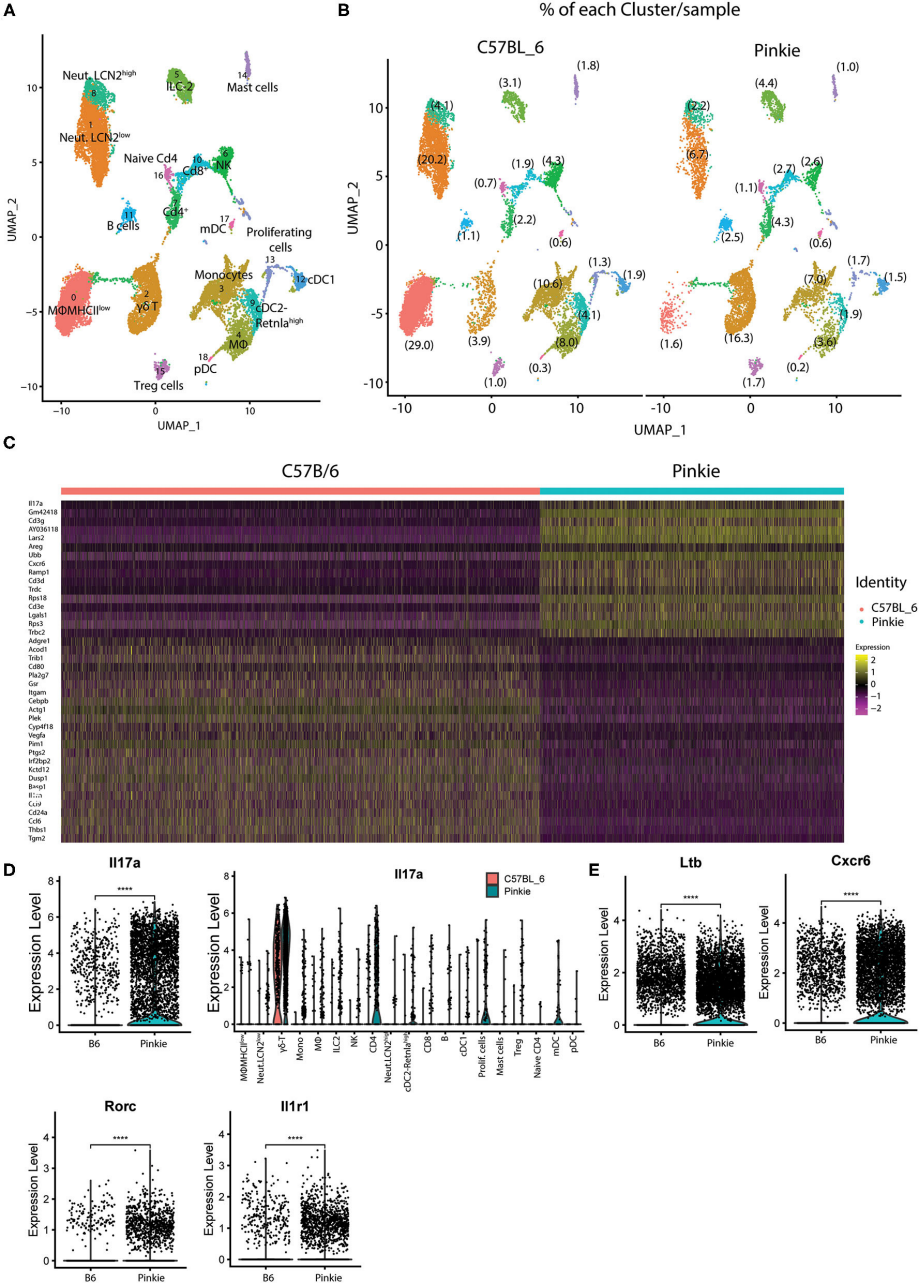
Single-cell RNA sequencing. Single-cell RNA sequencing (scRNA-seq) revealed differences in conjunctival immune cell populations between B6 and Pinkie. **(A)** UMAP of 19 distinct immune cell clusters in the conjunctiva generated from single-cell transcriptomic profiles of CD45^+^ cells using Seurat package V4.1.0. **(B)** UMAP comparing conjunctival immune cell clusters obtained by scRNA seq of CD45^+^ cells obtained from 8 mice female C57BL/6J and 8 Pinkie age 16 weeks. The percentage of the cells in each cluster is shown in parentheses and the cell count and percentage for clusters are provided in Table 1. **(C)** Heatmap of the top 50 differentially expressed genes in C57BL/6 and Pinkie conjunctival immune cells. Color of the heatmap based on the natural log of the normalized RNA expression; **(D)** Violin plots showing expression of IL-17a with expression in each cluster in the plots to the right. **(E)** Violin plots of γδT cell IL-17 signature genes *Ltb, Cxcr6, Rorc*, and *IL1rf* that have significantly higher expression in Pinkie vs. C57BL/6 (B6). ****p < 0.0001.

Inflammation has been found to cause ocular surface epithelial disease in dry eye. We performed droplet-based single-cell RNA sequencing (scRNA-seq) as an unbiased approach to compare immune cell types in the conjunctiva of WT and Pinkie strains. We constructed scRNA-seq libraries from CD45^+^ immune cells sorted from conjunctivas of normal WT and Pinkie (n=8 biological replicates/strain) and obtained transcriptomic profiles of these cells using the 10× Genomics platform. The scRNA-seq data analysis was performed using Seurat V4.1.0. After quality assessment, filtering standard pre-processing, and doublet exclusion, a total of 11,165 cells from B6 and 7,096 cells from Pinkie with 2,000 variable features were analyzed. Graph-based clustering using Seurat divided the cells into 19 clusters (Figure 2A) that were identified based on the expression of signature marker genes listed in Table 1 and shown in Supplementary Figure 1. The top 20 differentially expressed genes in each cluster are listed in Supplementary Table 2 (the entire list of differentially expressed genes in Supplementary Table 3). The major differences between the two strains are a decreased percentage of macrophages in cluster 0 and an increased percentage of γδ T cells in cluster 2 (Figure 2B, Table 1*)*. A heatmap of the top 50 differentially expressed genes between the strains is shown in Figure 2C and the complete list of differentially expressed genes is provided in the Supplementary Table 3. Il17a is the top differentially expressed gene. Violin plots in Figure 2D show significantly higher IL17a in Pinkie conjunctiva as compared to B6 predominantly produced by γδ T and conventional T cells which comprise 16.3 and 3.9 percent of the total cell population, respectively (Figure 2A). Pinkie also has increased IL-17f, predominantly produced by γδ T and conventional T cells (Supplemenatry Figure 2).

**Table 1:** Cluster identity.

		**C57BL_6**	**Pinkie**	**CIPR cluster identification**		
**Cluster**	**Name**	**Count (%)**	**Count (%)**	**Reference** **cell_type**	**reference_id**	**Percent_pos_** **correlation**
0	MΦMHCII^low^	3,239 (29.0)	181 (1.6)	Macrophage	MF.II-480hi.PC	60
1	Neutrophils-LCN2^low^	2,252 (20.2)	750 (6.7)	Granulocyte	GN.Thio.PC	100
2	γδ-T	434 (3.9)	1,820 (16.3)	γδ-T cell	Tgd.vg2+24alo.Th	93.33
3	Monocytes	1,180 (10.6)	782 (7.0)	Macrophage	MF.11c-11b+.Lu	90.47
4	MΦ	889 (8.0)	402 (3.6)	DC	DC.103-11b+24+.Lu	100
5	ILC2	349 (3.1)	492 (4.4)	ILC-2	ILC2.SI	100
6	NK cells	481 (4.3)	291 (2.6)	NK cell	NK.CD127-.SI	100
7	CD4^+^ T cells	249 (2.2)	484 (4.3)	T cell	T.4Mem49d+11a+.Sp.d30.LCMV	100
8	Neutrophils-LCN2^high^	454 (4.1)	247 (2.2)	Granulocyte	GN.Thio.PC	100
9	cDC2-Retnla^high^	460 (4.1)	209 (1.9)	DC	DC.11b+.AT.v2	100
10	CD8^+^ T cells	209 (1.9)	301 (2.7)	NK cell	NK.CD127-.SI	100
11	B cells	121 (1.1)	275 (2.5)	B cell	B.T2.Sp	100
12	cDC1	212 (1.9)	168 (1.5)	DC	DC.8-.Th	100
13	Proliferating cell	148 (1.3)	185 (1.7)	Pre-T cell	T.DPbl.Th	92.30
14	Mast cells	202 (1.8)	113 (1.0)	Mast cell	MC.Tr	98.24
15	Treg cells	113 (1.0)	186 (1.7)	Treg	ABD.TR.14w.B6	100
16	Naive CD4+	83 (0.7)	123 (1.1)	T cell	CD4.1h.LN	100
17	Migratory DC (mDC)	62 (0.6)	63 (0.6)	DC	DC.IIhilang-103-11b+.SLN	100
18	Plasma-cytoid DC (pDC)	28 (0.3)	21 (0.2)	DC	DC.pDC.8-.Sp	100
		11,165	7,093			

Significant between strain differences are also seen for expression of IL17 signature genes *Ltb* (25)*, Cxcr6* (26)*, Rorc* (25, 27, 28), and Il1r1 (29) among all cells (Figure 2E), and these are also significantly increased in Pinkie as compared to B6(Supplementary Figure 2).

**Figure 3:**
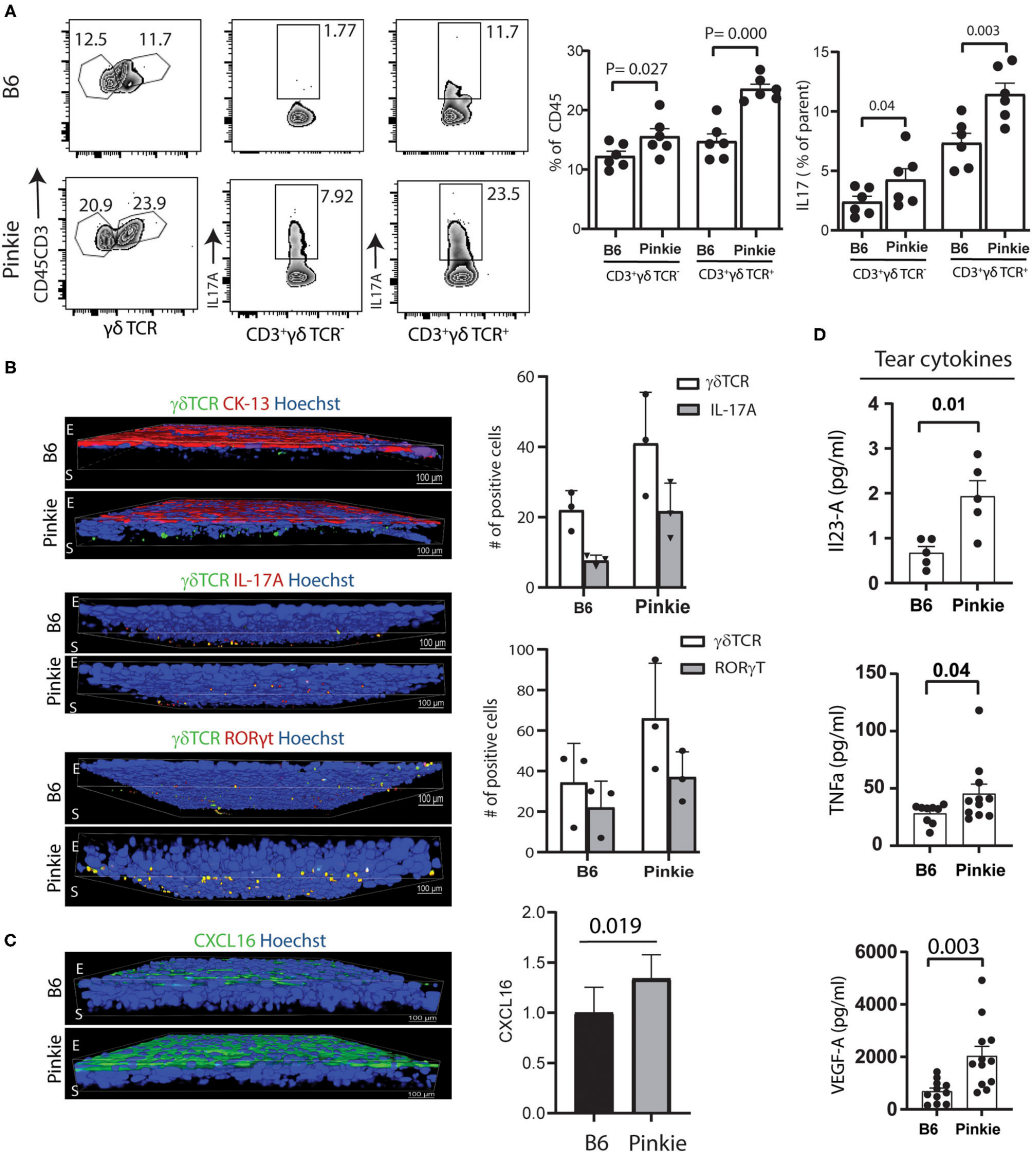
Increased γδ T cells in the Pinkie conjunctiva. **(A)** Flow cytometry of γδ T cell receptor positive cells (γδ TCR) left, IL-17A^+^CD3^+^ γδ TCR- center and IL-17A^+^CD3^+^γδTCR^+^ right [C57BL/6 (B6) strain top and Pinkie strain bottom] Bar graphs show the mean +/– SD of the percentage of cells in these groups (*n* = 6/group). **(B)** Confocal microscopy of whole-mount conjunctivas obtained from B6 and Pinkie (age 8–10 weeks) costained with antibodies specific for γδ TCR and conjunctival specific cytokeratin 13 (CK-13) (top), IL-17A (middle), and γδ T cell transcription factor RORγt (bottom) (*n* = 3 per group). The number of γδ T cells positive for IL-17A and RORγt are shown in the bar graphs to the right. Antibody details are provided in Supplementary Table 2. E, epithelium; S, stroma. **(C)** Confocal microscopy of whole-mount conjunctivas obtained from B6 and Pinkie (age 8–10 weeks) stained with CXCL16 antibody (left, *n* = 3). Minimal staining was observed in the B6 conjunctival. Comparison of CXCL16 expression level (fold change) in the conjunctiva measured by real-time PCR (right, *n* = 6). **(D)** Tear concentrations of IL-17 signature cytokines IL-12A/IL-23A (top) and TNF-a (middle), and angiogenic factor VEGF-A (bottom) measured by Luminex multiplex assay (*n* = 5–12/group, age 8–10 weeks).

Flow cytometry confirmed that Pinkie has significantly high IL-17 producing γδT cell receptor (TCR) negative (non- γδ T) and γδT cell receptor (TCR) positive CD3^+^T (γδ T) cells in the conjunctiva both in number and mean fluorescent intensity (MFI) (Figure 3A). Immunostaining of whole-mount conjunctivas shows an increased number of total, IL-17a^+^ and RORγt^+^ γδ TCR^+^ cells in the conjunctiva (Figure 3B). Minimal immunostaining for chemokine CXCL16, the ligand for CXCR6 that is expressed by γδT cells (26) is noted in the B6 corneal epithelium, but strong staining is seen in the Pinkie conjunctival epithelium and is accompanied by increased mRNA expression in the conjunctival epithelium (Figure 3C).

Increased concentrations of the IL-17 inducers IL-23 (30) and TNF-α (31, 32) as well as VEGF, a proangiogenic cytokine that promotes corneal neovascularization (33, 34) are found in Pinkie tears (Figure 3D).

### 9-cisRA Suppresses IL-17 Production by γδT Cells and Production of IL-23 by Monocytes

**Figure 4:**
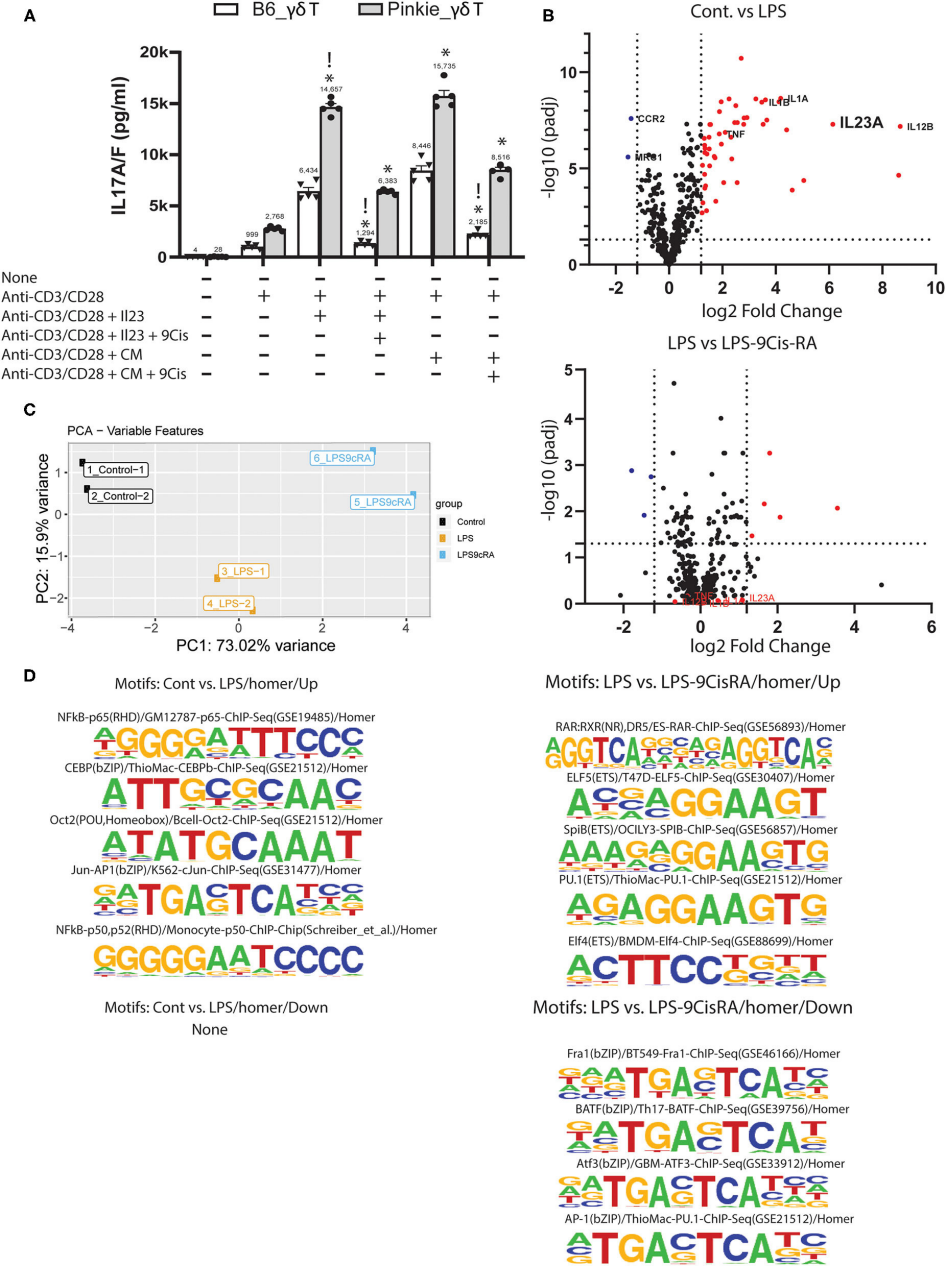
Suppressive effects of 9-cis retinoic acid. **(A)** IL-17A/F concentration in supernatants of cultured γδ T cells isolated with magnetic beads from C57BL/6 or Pinkie spleens. Cells are stimulated with anti-CD3/CD28 beads or beads plus recombinant IL-23 without or with addition of 100 μM 9-cis retinoic acid (RA). IL-17A/F is measured by ELISA. !*p* < 0.05 between treatment groups, *p < 0.05 between B6 and Pinkie strains. **(B)** Volcano plots showing the expression level of genes in monocytes cultured in media alone, media plus LPS or media plus LPS and 100 nM 9-cis RA. A mouse myeloid Innate Immunity NanoString array was used to evaluate gene expression. Dotted vertical lines indicate < or >1.5 log_2_ fold change and horizontal lines indicate genes with an adjusted *p* > 0.05. Red dots are genes that are significantly increased by LPS (top) or by LPS + 9-cis RA (bottom). γδ T inducers (TNF-a, IL-1a, IL-1b, and IL-23a) are stimulated by LPS and reduced by 9-cis RA. **(C)** ATAC seq: principal component analysis of peak sequences identified by ATAC seq in 3 experimental groups of cultured murine monocytes: control, cells stimulated with LPS and cells stimulated with LPS and 100 nM 9-cis RA (*n* = 2/group). **(D)** Sequence logos of transcription factor binding motifs that are found to be increased (up) or decreased (down) in the second group compared to the first group (top 4–5 motifs are shown for each group, except control vs. LPS where no decrease in motifs are found). Motifs are identified by the HOMER peak caller from databases of known motif sequences (20).

Based on our finding of increased γδT in the Pinkie conjunctiva, we evaluated if 9-cisRA suppresses IL-17 production by activated γδT cells in culture. γδT cells isolated from the spleen were stimulated with anti-CD3/CD28 beads with or without IL-23 and/or 9-cis RA. IL-17A/F was measured in the supernatant by ELISA. IL-17 release was higher in Pinkie γδT cells stimulated with beads or beads + IL-23 (Figure 4A). 9-cis RA significantly reduced the supernatant IL-17 concentration in cells from both strains, although the suppressive effect was greater in the B6 cells (74 vs. 46% in bead+IL-23 stimulated cells). The majority of myeloid cells in the conjunctiva express RXRα and when stimulated with LPS they produce factors known to stimulate IL-17 production by γδ T cells (8). We compared the stimulatory activity of conditioned media from LPS-treated monocytes to recombinant IL-23 on IL-17 production by γδ T cells and found they are equivalent (Figure 4A). Furthermore, treatment of LPS-stimulated cultured monocytes with 9-cisRA significantly reduced stimulatory activity of their conditioned media (Figure 4). Consistent with these findings, we found that both genes encoding the IL-23 heterodimer (Il23a and Il12b), as well as other γδT inducing cytokines Il1α, Il1β, and Tnf-α are significantly upregulated in LPS-stimulated cultured monocytes measured in a Nanostring array (Figure 4B, top), and these are suppressed by addition of 9-cis RA to the culture media (Figure 4B bottom).

Retinoic acid is known to cause epigenetic changes that can affect transcription factor binding and gene transcription (35). We performed ATAC seq on cultured monocytes to determine if 9-cis RA treatment changes the number of open transcription factor (TF) binding motifs in LPs-stimulated cultured monocytes. The PCA plot in Figure 4C shows marked differences in peak region sequences in areas of open chromatin between control, LPS-treated and LPS+9-cis RA treated cells. LPS treatment significantly increased the number of TF motifs regulating transcription of inflammatory cytokines, including NFkB and Jun-AP-1 (Figure 4D, top left), but did not reduce the number of any known motifs (Figure 4D, bottom left). Compared to LPS treatment alone, 9-cis RA+LPS increased the number of 5 known motifs, including RAR:RXR Figure 4D (top right), and decreased the number of 4 motifs, including AP-1 Figure 4D (bottom right). AP-1 is a key transcription factor for Il23a and other inflammatory mediators (36).

Taken together, these findings indicate that RXRα suppresses the production of IL-17 by activated γδ T cells and the production of monocyte cytokines known to stimulate IL-17 production by γδT cells.

### Differential Pathway Analysis Reveals Increased IL-17 Signaling in *Pinkie*

**Figure 5:**
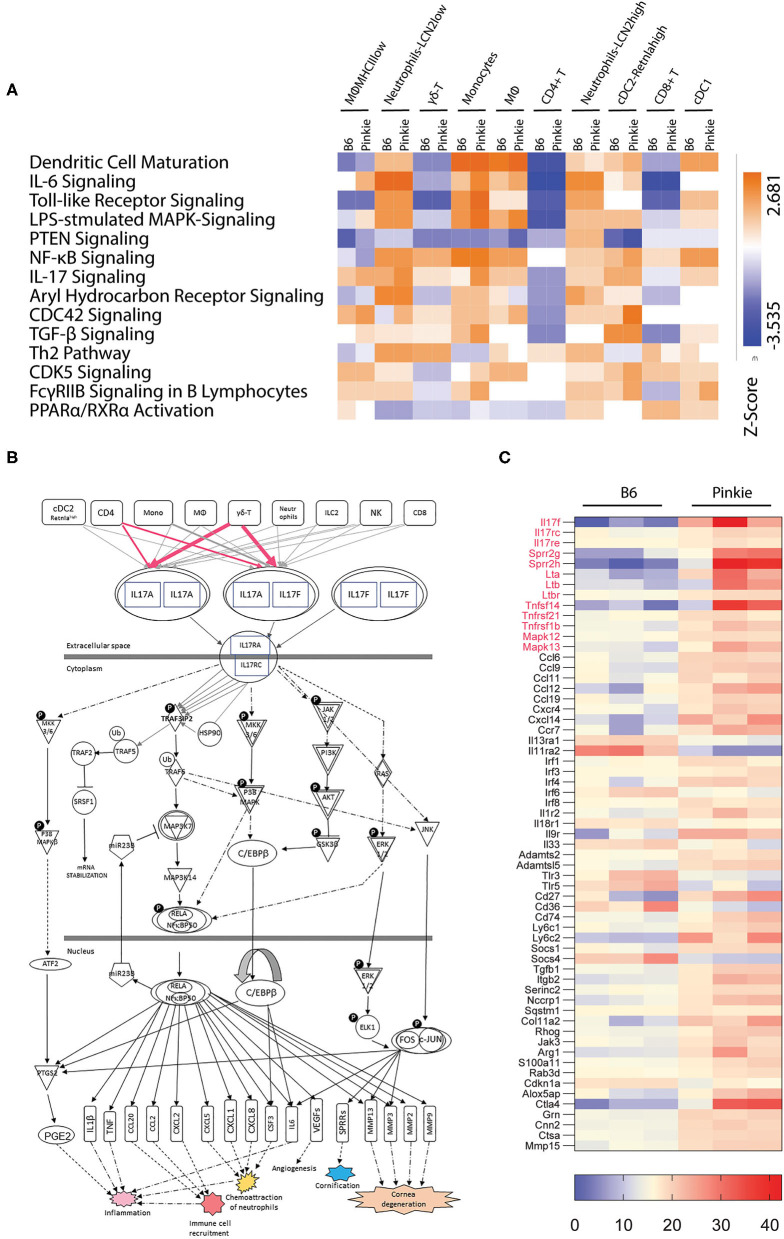
Pathway analysis. **(A)** Heatmap of canonical pathways showing significant differences between strains and cell clusters was generated by Qiagen Ingenuity Pathway Analysis. This analysis identified the pathways from the Ingenuity Pathway Analysis library of canonical pathways that were most relevant to the data set. Molecules from the data set that had an adjusted *p* < 0.05 and were associated with a canonical pathway in the Ingenuity Knowledge Base were considered for the analysis. The significance of the association between the data set and the canonical pathway was measured in two ways: (1) A ratio of the number of molecules from the data set that map to the pathway divided by the total number of molecules that map to the canonical pathway is displayed; and (2) A right-tailed Fisher's Exact Test was used to calculate a *p*-value determining the probability that the association between the genes in the dataset and the canonical pathway is explained by chance alone. IL-17 signaling and PPARα/RXRα activation pathways are among the pathways identified with significant differences. **(B)** IL-17 signaling pathway network showing the relationship between molecules generated with Qiagen Ingenuity Pathway Analysis with modification. All connections are supported by at least one reference from the literature, from a textbook, or from canonical information stored in the Ingenuity Knowledge Base. Lines and arrows between nodes represent direct (solid) or indirect (dashed) interactions between gene products and are displayed by cellular localization (extracellular space, plasma membrane, cytoplasm, or nucleus). Rectangles are cytokines and cytokine receptors, triangles are phosphatases, concentric circles are groups or complexes, diamonds are enzymes and ovals are transcriptional regulators or modulators. P, phosphorylation; U, ubiquitination. **(C)** Heatmap of differentially expressed genes in conjunctival bulk RNA seq between C57BL/6 (B6) and Pinkie strains that includes IL-17 pathway associated genes in red and other innate inflammatory mediators. All the genes passed through the Benjamini-Hochberg procedure to exclude false discovery; the selected genes had an adjusted *p* > 0.05. Each row represents a specific gene, the right column represents the Pinkie strain and left column represents B6.

RXRα nuclear receptor regulates the expression of an array of inflammatory mediators. We used QIAGEN Ingenuity Pathway Analysis (IPA) tool to identify significant differences (*p* < 0.05) between B6 and Pinkie in inflammatory signaling pathways generated from the scRNAseq data. These pathways grouped by strain and cell type are displayed in the heatmap shown in Figure 5A. The greatest differences are seen in neutrophils, myeloid (macrophage and monocyte) and cDC2 cells and include IL-6, LPS-stimulated MAPK, NFkB, IL-17, and PPARα/RXRα signaling pathways that contain mediators relevant to dry eye pathogenesis (8, 37–42). PPARα/RXRα signaling was significantly reduced in MHCII low macrophages and monocytes.Two other pathways, CDC42 and CDk5, have been implicated in NLRP3 inflammasome activation (43, 44). The annotated IL-17 signaling pathway generated with IPA (Figure 5B) contains downstream signaling pathways (MAPK and NFkB) that stimulate expression of cytokines that induce IL-17 production by γδT cells, as well as IL-17 inducible mediators (e.g., matrix metalloproteases, SPRR2) that are involved in the development of the cornea and conjunctival epithelial disease of dry eye.

The conjunctiva is a mucosal tissue composed of epithelial, stromal and immune cells that express IL-17 receptors and are potential IL-17 targets (45). To determine if IL-17 related genes/pathways are increased in the whole conjunctiva in Pinkie, we compared expression profiles generated from bulk RNA seq performed on whole conjunctival lysates harvested from B6 and Pinkie (Figure 5C). Similar to the scSeq performed on immune cells, we found IL-17a and IL-17f together with IL-17 receptor (IL-17rc) to be among of the top differentially expressed genes (adj *p* < 0.02) with increased expression in Pinkie (Figure 4C). There is also increased expression of other IL-17 signaling pathway associated genes, including cornified envelope precursor genes Sprr2g and Sprr2h (46, 47), p38 Mapks [Mapk12 (p38 gamma), and Mapk13 (P38 delta)], and chemokine CCL6. Various other inflammatory mediators and signaling molecules (e.g., Tlr3, Tlr5) are also increased. Taken together, this data indicates that RXRα suppresses the production of IL-17 by γδ T cells and that IL-17 can exert protean influence on epithelial and immune cells on the ocular surface.

### IL-17 Neutralization Suppresses the Development of Ocular Surface Disease in Pinkie Bone Marrow Chimeras Exposed to Desiccating Stress

**Figure 6:**
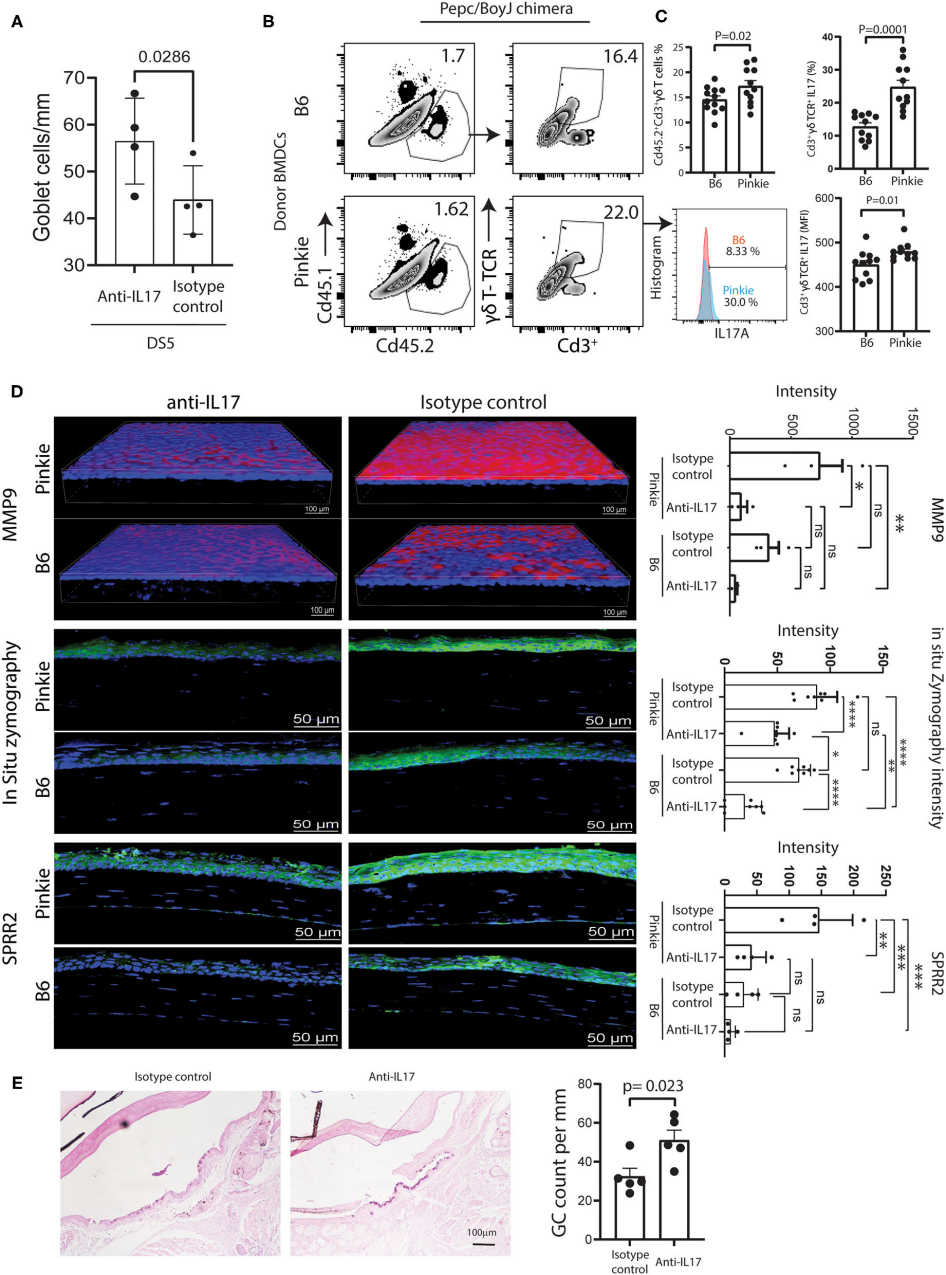
Comparison of γδT and dry eye signs in bone marrow chimeras. **(A)** Conjunctival goblet cell number in C57BL/6 mice exposed to desiccating stress (DS) for 5 days (DS5) with or without systemic treatment with anti-IL-17 neutralizing antibody or isotype control as described in the methods. **(B)** Representative flow cytometry plots of the donor (CD45.2^+^) and the recipient (CD45.1^+^) bone-marrow derived cells (left) and γδ TCR high and low CD3^+^ T cells in the conjunctivas of Pepc/BoyJ recipient (host) chimeric mice (age 8–10 weeks) reconstituted with B6 or Pinkie bone marrow after 5 days of desiccating stress. The method of chimera creation is provided in Supplementary Figure 3. **(C)** Top left: bar graph shows the percentage of CD45.2^+^CD3^+^γδTCR^+^ in the recipient conjunctiva (*n* = 11/group). Bottom left: histogram of percentage of IL-17^+^ cells from CD45.2^+^CD3^+^γδTCR^+^ gate in representative sample. Right: mean +/– SD percentage (top) and mean fluorescent intensity (bottom) of IL-17A^+^CD3^+^γδ TCR^+^ cells in the conjunctiva of chimeras (*n* = 11/group). **(D)** Confocal microscopy of whole-mount conjunctivas or cryosections obtained from B6 and Pinkie bone marrow chimeras created as shown in Supplementary Figure 3 with or without systemic treatment with anti-IL-17 neutralizing antibody or isotype control as described in the methods and exposed to DS for 5 days stained with an antibody specific for MMP-9 (top), evaluated for *in situ* gelatinase (zymography) activity in cryosections (middle), or stained with polyclonal antibody to cornified envelope precursor SPRR2 (bottom) (*n* = 3 per group). Bar graphs to the right show mean ± SD fluorescent intensity of the fluorochrome/fluorescent gelatin measured by Nikon Elements software (*n* = 3/group). **(E)** Conjunctival goblet cell number in Pinkie donor bone marrow chimeric mice exposed to desiccating stress (DS) for 5 days (DS5) with or without systemic treatment with anti-IL-17 neutralizing antibody or isotype control as described in the methods. Representative photomicrographs of periodic acid-stained sections for each treatment group (left) and graph of mean ± SD of goblet cells/mm (*n* = 5). Some goblet cells in the control group appear entrapped in the epithelium as previously reported (24).

We previously reported IL-17 causes corneal barrier disruption in mice subjected to experimental desiccating stress (DS) by stimulating expression of metalloproteinases (MMP-3 and MMP-9) that lyse tight junction proteins in the apical corneal epithelium (39). In that study, mice treated with anti-IL-17 had significantly less barrier disruption and reduced MMP-9 expression, MMP-9 immunostaining and gelatinase activity. In a previously unpublished experiment, we also found IL-17 neutralization prevented DS-induced conjunctival goblet loss (Figure 6A).

We hypothesized that bone marrow chimeras created with Pinkie donor cells would produce greater ocular surface disease than those created with B6 donor cells because reduced RXRα signaling in Pinkie will lead to an increased infiltration of the conjunctiva by donor γδT cells. Bone chimeras created by a previously reported method (17) and summarized in Supplementary Figure 3 were exposed to DS for 5 days (17). Chimeric mice were treated with either anti-IL-17 or isotype control antibodies every 2 days starting 2 days prior to initiating DS. After 5 days of DS, the percentages of conventional T cells, γδT cells, and IL-17+ cells in the conjunctiva were evaluated by flow cytometry and measures of dry eye disease, including corneal MMP-9 immunoreactivity and gelatinase activity (*in situ* zymography), and conjunctival goblet cell number. Pinkie donor chimeras were found to have a greater percentage of γδT cells and a greater percentage and MFI of IL-17^+^γδT cells (Figures 6B,C). In both chimeras, γδT cells are the major IL-17 producers. Among IL-17+ cells, the ratio of γδT cells/conventional T cells is 89% in B6 chimeras and 97% in Pinkie chimeras (*p* < 0.0001 for both). MMP-9 immunoreactivity is significantly lower in anti-IL-17 treated than control-treated Pinkie chimeras, and *in situ* gelatinase activity was lower in the anti-IL17 treated Pinkie and B6 chimeras (Figure 6D). Anti-IL-17 treatment also reduced MMP-9 and SPPR2 immunostaining in the Pinkie corneal epithelium and gelatinase activity in the corneal epithelium of both strains (Figure 6D). Conjunctival goblet cell density was significantly higher in the anti-IL-17 treated chimeras (Figure 6E).

Taken together these data show that reduced RXRα signaling enhances migration of γδT cells to the conjunctiva in dry eye and that IL-17 produced by these cells causes corneal and conjunctival epithelial disease.

### Pinkie Develops Corneal Neovascularization, Opacification, and Ulceration With Aging

**Figure 7:**
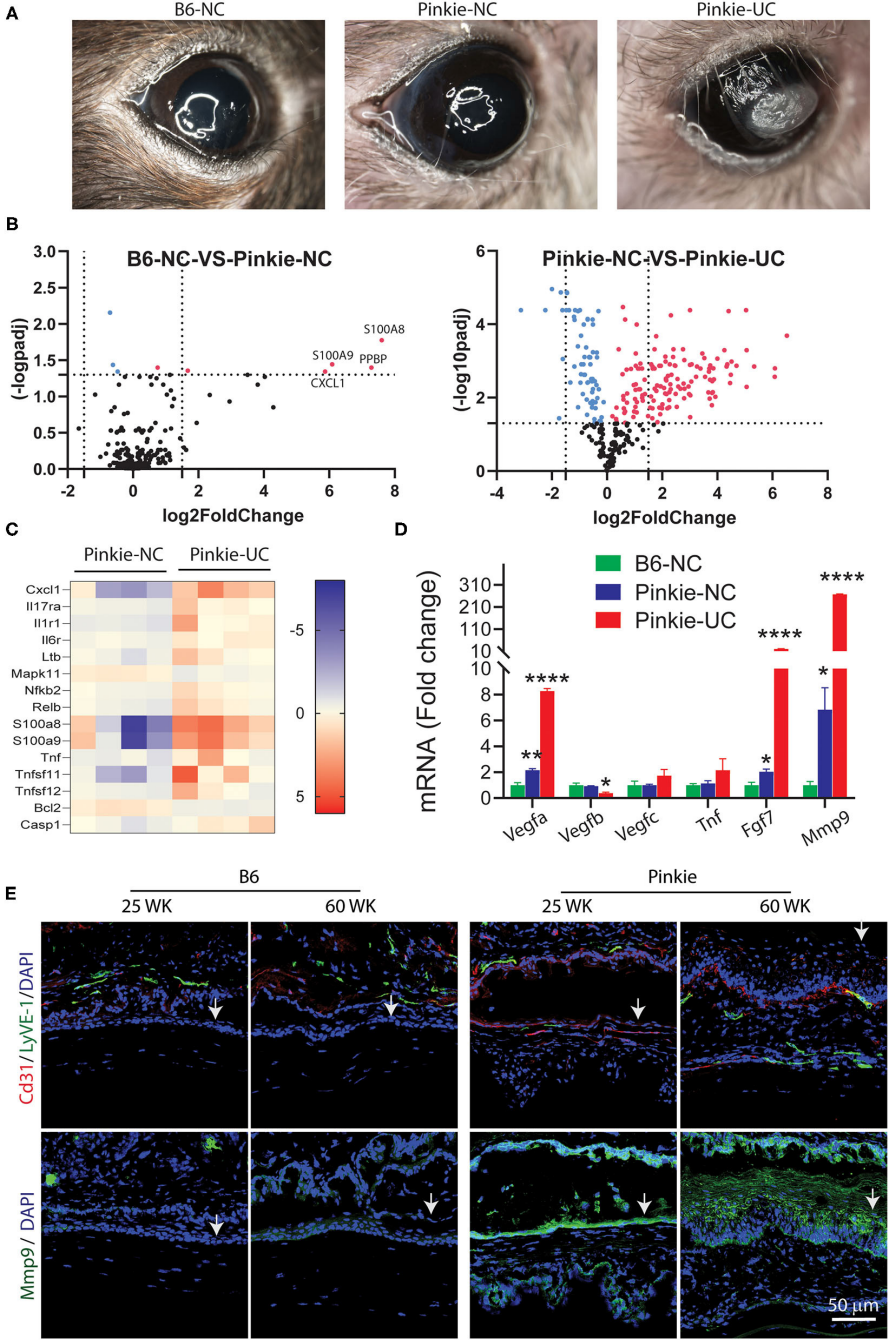
**(A)** Appearance nonulcerated (NC) C57BL/6 (B6) and NC and ulcerated (UC) Pinkie corneas in 40–50-week-old mice. **(B)** Volcano plots of differentially expressed genes in corneas of NC B6 and Pinkie (left) and NC and UC Pinkie (right) detected by a mouse myeloid Innate Immunity Nanostring array. Dotted vertical lines indicate < or >1.5 log_2_ fold change and horizontal lines indicate genes with an adjusted *p* > 0.05. Red dots are genes that are significantly increased in Pinkie NC vs. B6 NC (left) or in Pinkie UC vs. Pinkie NC (left). Labeled genes in the left plot are found in the IL-17 signaling pathway. **(C)** Heat maps generated from the NanoString array of IL-17 pathway genes. **(D)** Fold change of expression level of factors involved in the pathogenesis of corneal vascularization (*Vegfa, Tnf* , *Fgf7)* or corneal ulceration (*Mmp9*) measured by RT-PCR. Values are mean ± SD (*n* = 4/sample). **(E)** Immunostaining of blood (CD31) and lymphatic (LyVE-1) endothelial cell markers in 25- and 60-week-old B6 and Pinkie corneas. Arrows indicate corneal epithelium. (*n* = 4/sample). *p < 0.01; **p < 0.001; ****p < 0.0001.

We observed the Pinkie strain develops corneal opacification, neovascularization and ulceration with aging (Figure 7A). Corneal opacity and vascularization were noted in 14% of 144 Pinkie eyes compared to only 2% of 100 B6 eyes. We compared gene expression profiles in NanoString myeloid innate immunity arrays performed on whole cornea lysates prepared from 45 to 60 week old B6 or Pinkie with normal-appearing corneas (NC) or from Pinkie with ulcerated corneas (UC). A volcano plot shows 4 genes with significantly elevated expression in Pinke NC compared to B6 NC (Figure 7B, top left). Significantly increased expression of numerous genes are noted when comparing normal and ulcerated Pinkie corneas (Figure 7B, right). Included among the significantly differentially expressed genes in the Pinkie ulcerated cornea are the IL-17 signaling pathway genes displayed in the heatmap (Figure 7C). These include γδT inducers (i.e., *Ltb, Tnf, Nfkb2, Relb*) and *Il17ra*.

Expression levels of several factors that promote corneal vascularization (*Vegfa, Fgf7*) and ulceration (*Mmp9*) measured by PCR are significantly increased in the Pinkie UC (Figure 7D). Interestingly, expression of *Vegfb* which has trophic activity on corneal nerves was reduced in the Pinkie UC (48). Consistent with these findings is increased immunoreactivity of blood and lymphatic endothelial markers CD31/LYVE-1 and MMP-9 in the corneal epithelium of older Pinkie compared to similarly aged B6 (Figure 7E). These findings suggest that dry eye combined with chronic elevation of pro-angiogenic and proteolytic factors in Pinkie promotes corneal vascularization, opacification and ulceration.

## Discussion

This study investigated the mechanism for developing dry eye disease in the Pinkie strain with a loss of function RXRα gene mutation. Using scRNA-seq as an unbiased approach to investigate the conjunctival immune cell population, we discovered a four-fold greater percentage of conjunctival γδ T cells with higher expression of IL-17 and other γδ T cell signature genes. The sequencing findings are confirmed by flow cytometry and confocal microscopy that shows these cells are located in the stroma beneath the conjunctival epithelium. The Pinkie strain developed accelerated signs of dry eye disease in the cornea and conjunctiva. To determine the pathogenicity of Pinkie γδ T cells we created bone marrow chimeras using Pinkie donor cells and found a significant reduction in corneal and conjunctival disease in the group receiving IL-17 neutralizing antibody.

Our group and others have found that IL-17 is involved in the pathogenesis of the corneal epithelial disease of dry eye (37, 39). IL-17 stimulates MMP expression by the corneal epithelium, as well as neutrophil recruitment and activation (39, 49). MMP-9 disrupts the corneal epithelial barrier via lysis of tight junction proteins in the apical epithelium that results in accelerated desquamation (50). Conjunctival goblet cell loss in dry eye can develop from cytokine-mediated apoptosis or altered differentiation with entrapment of goblet cells by abnormally differentiated epithelium with increased expression of cornified envelope precursors such as SPRR2 which is induced by IL-17 (46, 47).

Our previously reported studies found antibody neutralization of IL-17 significantly reduces corneal barrier disruption measured by OGD permeability in the desiccating stress model of dry eye (39). While performing those studies, we also found anti-IL-17 prevented desiccation-induced conjunctival goblet cell loss. Studies reported by others have also found that IL-17 produced by Th17 cells causes cornea and conjunctival disease (37, 51). IL-17 is primarily produced by CD4^+^ T cells and γδ T cells. IL-17 was detected in CD4^+^ T cells by flow cytometry in previous studies using the DS dry eye model, but most did not evaluate IL-17 production by conjunctival γδ T cells. Increased expression of IL-17 was noted in the conjunctival epithelium of patients with Sjögren syndrome keratoconjunctivitis sicca, but the cellular source was not determined (52). γδ T cells were the second most prevalent population of intraepithelial lymphocytes in the mouse conjunctiva (53), and Coursey et al. reported that IL-17 is produced by γδ T cells in the conjunctiva of the NOD mouse strain that develops KCS and is used as a model of SS (54). This study suggests that conjunctival γδ T cells are another source of IL-17 and that IL-17 expression in these cells is regulated by the RXRα nuclear receptor. γδ T cells are found in many mucosal surfaces and can be activated in a non-antigen-specific manner by a variety of PAMPs and conceivably to desiccating stress that activates the same signaling pathways as microbial products (55).

The RXR nuclear receptor family regulates the transcription of numerous genes involved in immune function, cell differentiation and homeostasis. RXRα may function as a homodimer or a heterodimer with partner receptors (PPARγ and the vitamin D receptor) that have been found on the ocular surface (56, 57). The ocular surface is a retinoid-rich environment (8). Besides the retinol form of vitamin A in tears that is converted to the natural ligand 9-cis RA by aldehyde dehydrogenases in myeloid and epithelial cells on the ocular surface (6), nutritional ligands such as vitamin D, the omega-3 fatty acid DHA in fish oil and oleic acid in olive oil can bind certain RXR dimeric partners (3).

**Figure 8:**
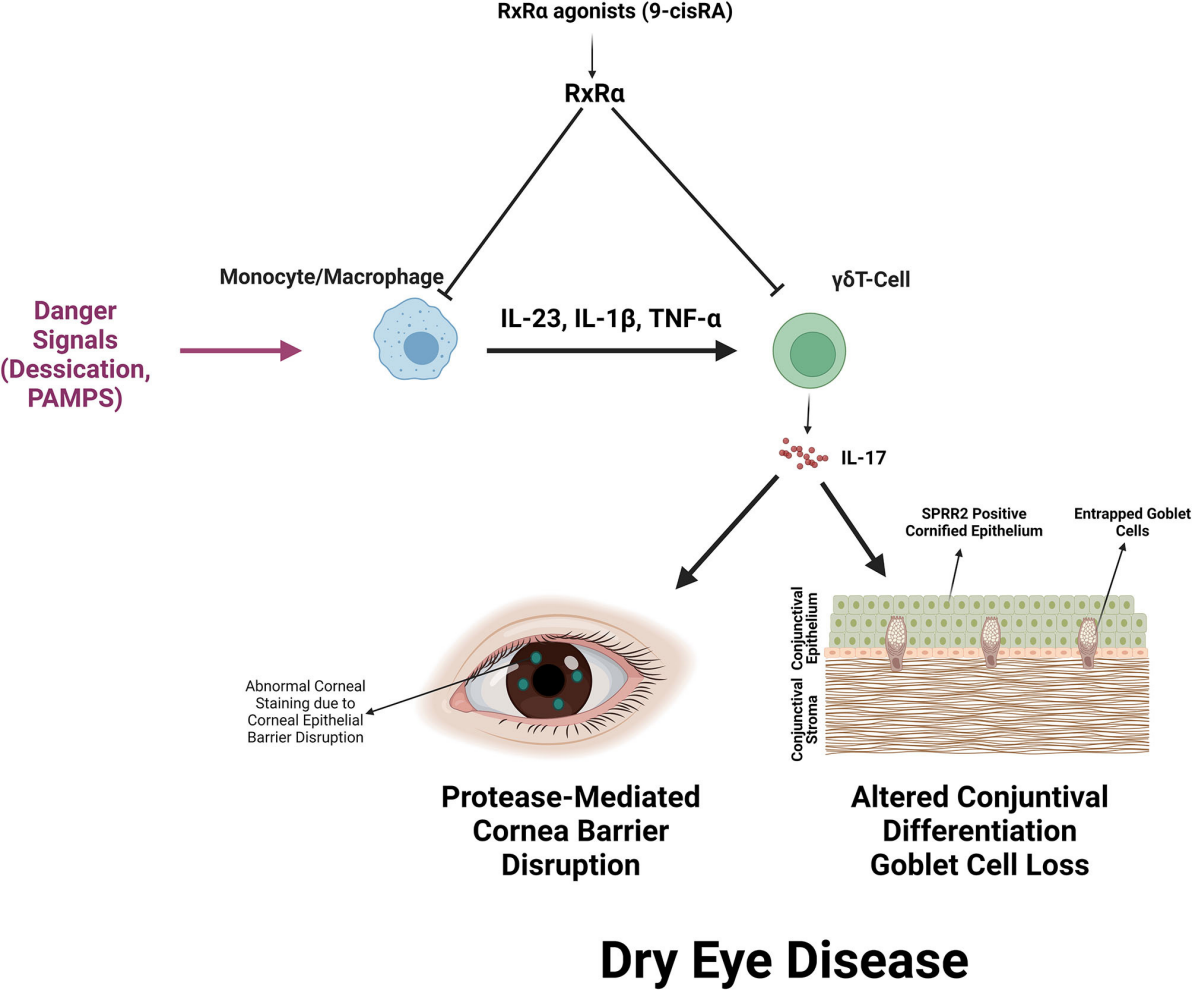
Summary of RXRα mediated suppression of IL-17 production by γδ T cells and IL-17 mediated dry eye disease. RXRα suppresses the production of IL-17 inducers (IL-23, IL-1β, and TNF-α) by myeloid cells and directly suppresses IL-17 production by activated γδ T cells. IL-17 promotes corneal barrier disruption, increased expression of the cornified envelope precursor SPRR2 that decreases epithelial lubricity and seals goblet cell openings and reduction in mucin-filled conjunctival goblet cells.

We previously reported that the majority of CD11b+ myeloid cells are RXRα positive and respond to retinoic acid (8). The discovery of increased IL-17 producing γδ T cells in the Pinkie strain indicates RXRα is also an important regulator of IL-17 production by γδ T cells. The synthetic retinoid AM80 was found to suppress IL-17 production by γδ T cells stimulated with anti-CD28 antibody and a cytokine cocktail of IL-23 and IL-1β (30). We found 9-cisRA suppressed IL-17 production by > 70% in cultured γδ T cells stimulated by CD28 beads or beads plus IL-23. In addition to direct suppression of γδ T cells, was also found 9-cisRA suppresses the expression of γδ T cell inducers (IL-23, IL-1, TNF-α) by cultured monocytes and we previously reported reduced levels of IL-1β and IL-23β in supernatants of 9-cis RA treated monocytes (8). Monocyte conditioned media has stimulatory activity equivalent to recombinant IL-23, but this was significantly reduced in monocytes cultured with 9-cis RA. We also found that 9-cis RA decreases the number of open AP-1 transcription factor binding motifs detected by ATAC seq. Both AP-1 and NFkB pathways are involved in stimulated IL-17 expression by γδ T cells (25, 58). Figure 8 summarizes the primary and secondary suppressive activity of 9-cis RA on the production of IL-17 by γδ T cells.

There are several weaknesses of this study. We performed single-cell profiling on conjunctival immune cells because it is difficult to obtain a sufficient number of donor cells from the cornea. It is possible the corneal pathology results from IL-17 produced by conjunctival γδ T cells, but IL-17 producing γδ T cells have been found to infiltrate the cornea following epithelial trauma (34, 59). We performed ATAC seq on monocytes which demonstrated the epigenetic effects of 9-cis RA has on these cells. Our discovery that RXRα suppresses IL-17 production by γδ T cells is rationale for evaluating epigenetic activity of 9-cis RA on these cells in the future.

The findings of this study suggest that RXRα retinoid signaling suppresses activation of dry eye disease-inducing IL-17 producing conjunctival lymphocytes under homeostatic conditions. Additional studies will be needed to determine if this signaling pathway is relevant in human dry eye. This signaling may be reduced in aqueous tear deficient dry eye due to reduced secretion of retinol into tears by dysfunctional lacrimal glands. Additionally, we have reported decreased aldehyde dehydrogenase expression in the conjunctiva in dry eye that could result in decreased RA synthesis (8). Strategies that maintain the ocular surface retinoid axis in dry eye may prevent IL-17 induced epithelial pathology.

## Data Availability Statement

Data presented and codes used for data analysis are available at these sites. R codes: https://github.com/jehanalam82/Increased-dry-eye-disease-inducing-T17-cells-in-the-RXR-mutant-mouse-.git. Single cell sequencing: https://singlecell.broadinstitute.org/single_cell/study/SCP1703, https://singlecell.broadinstitute.org/single_cell/study/SCP1614. NanoString and ATAC-sequencing: https://www.ncbi.nlm.nih.gov/geo/query/acc.cgi?acc=GSE192960.

## Ethics Statement

The animal study was reviewed and approved by Baylor College of Medicine IACUC.

## Author Contributions

JA, SP, and RR were involved in conception and design of the study. JA, GY, SP, CP, RS, and ZY were involved in data acquisition. JA, SP, GY, RS, NB, RR, DL, and CP were involved in data analysis and interpretation. JA, GY, RR, and SP drafted the manuscript. All authors reviewed and revised the manuscript critically for important intellectual content and approved the submitted version.

## Funding

This work was supported by NIH Grant EY11915 (SP), EY026893-04S1 (CP), NIH Core Grant EY002520, the Cytometry and Cell Sorting Core at Baylor College of Medicine (BCM) with funding from the CPRIT Core Facility Support Award (CPRIT-RP180672), the NIH grant (CA125123), and the assistance of Joel M. Sederstrom. Single Cell Genomics Core at BCM partially supported by National Institutes of Health (NIH) shared instrument grants (S10OD018033 and S10OD023469), and the BCM Genomic & RNA Profiling Core (GARP) [P30 Digestive Disease Center Support Grant (NIDDK-DK56338) and P30 Cancer Center Support Grant (NCI-CA125123), NIH S10 grant (1S10OD02346901)]. Additional support includes an unrestricted grant from Research to Prevent Blindness, New York, NY (SP), The Hamill Foundation, Houston, TX (SP) and the Sid W. Richardson Foundation, Ft Worth, TX (SP). Lions Eye Bank of Texas, Houston TX (JA), and Knights Templar Eye Foundation, Flower Mound, TX (JA).

## Conflict of Interest

The authors declare that the research was conducted in the absence of any commercial or financial relationships that could be construed as a potential conflict of interest.

## Publisher's Note

All claims expressed in this article are solely those of the authors and do not necessarily represent those of their affiliated organizations, or those of the publisher, the editors and the reviewers. Any product that may be evaluated in this article, or claim that may be made by its manufacturer, is not guaranteed or endorsed by the publisher.

## Supplementary Material

Supplementary Figure 1 UMAP feature plots of highly expressed genes in each cluster (cluster identity in parentheses) identified in scRNA-seq, except cluster 1 where expression of Lcn2 is low.

Supplementary Figure 2 Violin plots for IL17f with expression in each cluster in the plot to the right (top) along with other IL-17 signature genes *Ltb, Cxcr6, Rorc*, and *IL1rf* in cell clusters identified in scRNA-seq in Pinkie and C57BL/6 strains.

Supplementary Figure 3 Generation of bone marrow chimeric mice. Bone marrow ablation in Pepc/BoyJ recipient (host) mice was accomplished with ^137^Cs irradiation with 1,300 cGy, followed by intraorbital injection of 2 × 10 ^∧^ 6 bone marrow cells from wild type B6 or Pinkie donors. Two weeks after receiving donor cells, chimeric mice were exposed to desiccating stress for 5 days to create dry eye and the presence of donor bone marrow-derived cells was identified by flow cytometry performed on conjunctival samples. The representative scatter plot shows the endogenous (CD45.1) or transplanted (CD45.2) immune cells in the conjunctiva. The phenotype of CD45.2^+^ cells shown in the gate was further characterized.

Supplementary Table 1 Antibodies in this study.

Supplementary Table 2 Top differentially expressed genes in cell clusters.

Supplementary Table 3 Differentially expressed genes per cell cluster.
