# Supplementary figures and images for: IL-17 Producing Lymphocytes Cause Dry Eye and Corneal Disease With Aging in RXRα Mutant Mouse
Source: Front Med (Lausanne). 2022 Mar 23;9:849990. doi: 10.3389/fmed.2022.849990 (PMC8983848; doi:10.3389/fmed.2022.849990)

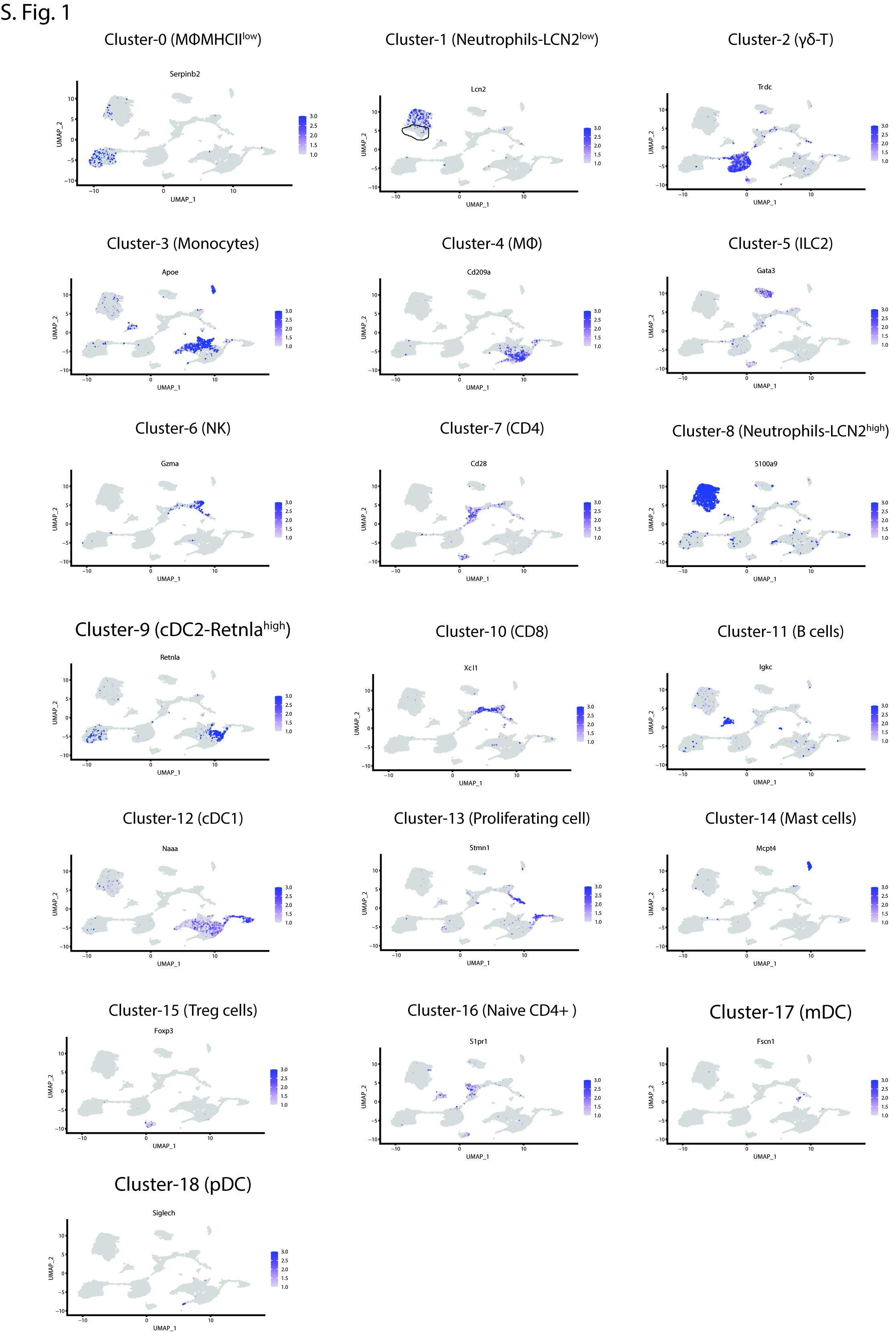

Supplement: Supplementary Figure 1 — UMAP feature plots of highly expressed genes in each cluster (cluster identity in parentheses) identified in scRNA-seq, except cluster 1 where expression of Lcn2 is low. [file Image_1.tif]

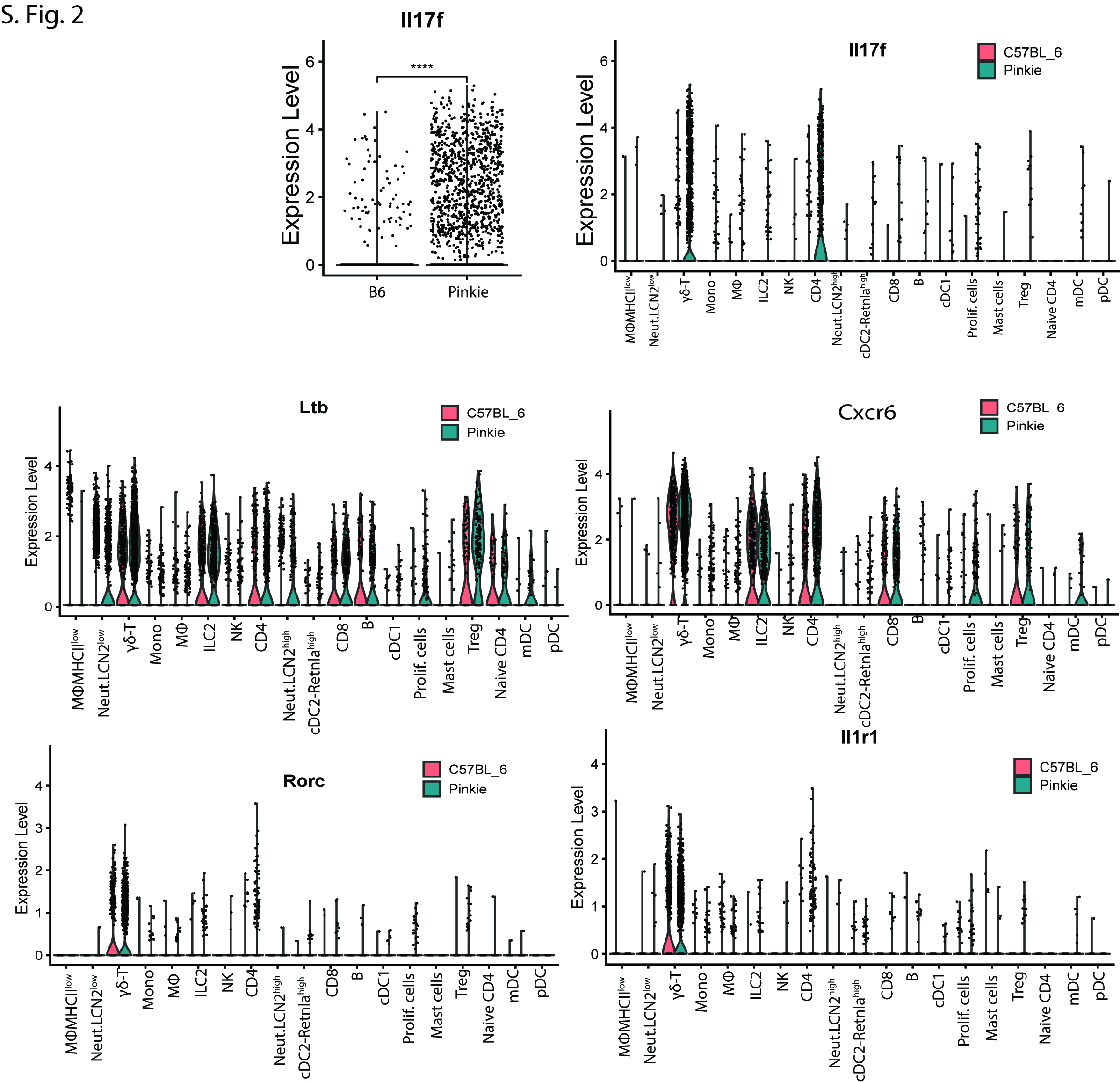

Supplement: Supplementary Figure 2 — Violin plots for IL17f with expression in each cluster in the plot to the right (top) along with other IL-17 signature genes Ltb, Cxcr6, Rorc, and IL1rf in cell clusters identified in scRNA-seq in Pinkie and C57BL/6 strains. [file Image_2.tif]

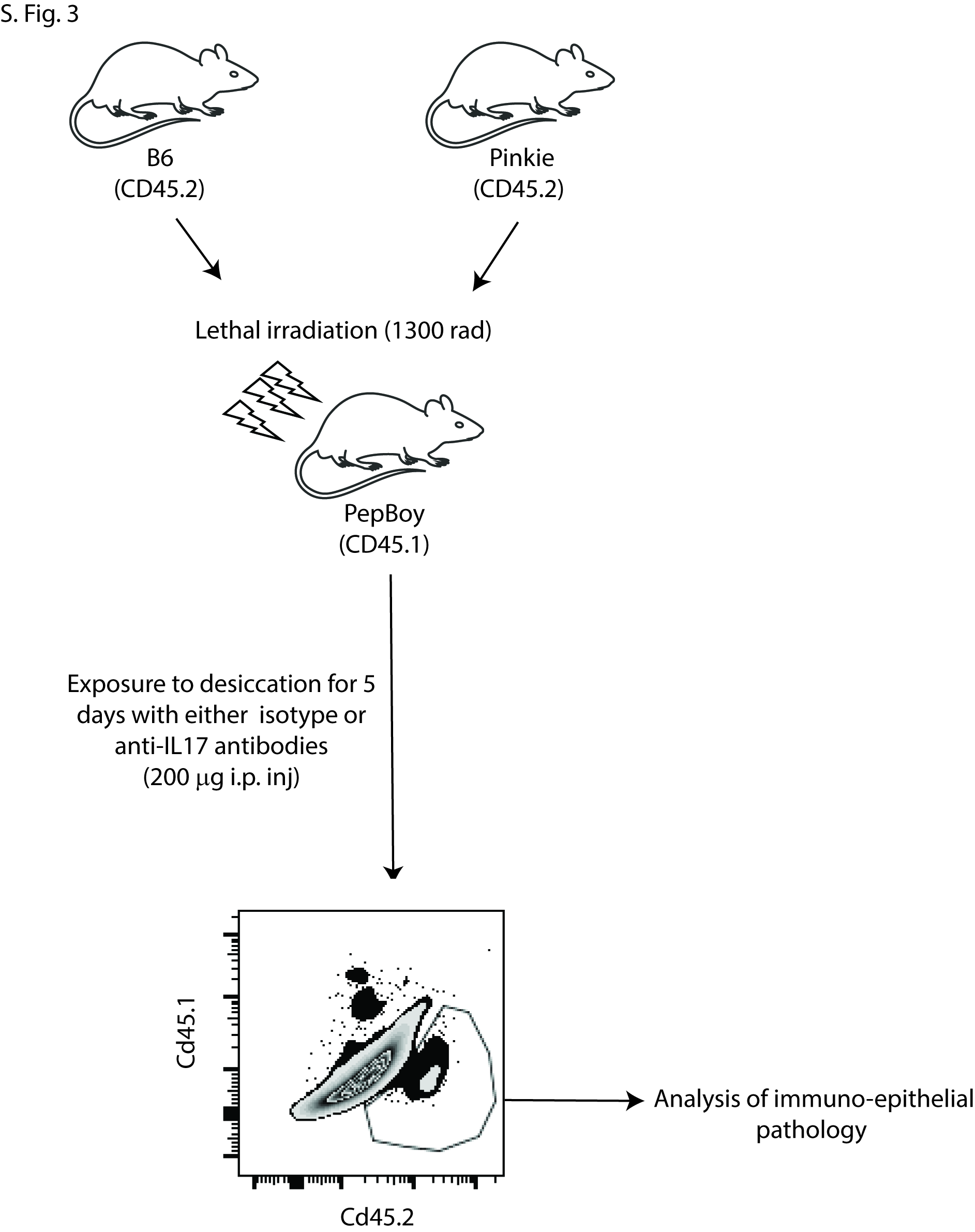

Supplement: Supplementary Figure 3 — Generation of bone marrow chimeric mice. Bone marrow ablation in Pepc/BoyJ recipient (host) mice was accomplished with 137Cs irradiation with 1,300 cGy, followed by intraorbital injection of 2 × 10 ∧ 6 bone marrow cells from wild type B6 or Pinkie donors. Two weeks after receiving donor cells, chimeric mice were exposed to desiccating stress for 5 days to create dry eye and the presence of donor bone marrow-derived cells was identified by flow cytometry performed on conjunctival samples. The representative scatter plot shows the endogenous (CD45.1) or transplanted (CD45.2) immune cells in the conjunctiva. The phenotype of CD45.2+ cells shown in the gate was further characterized. [file Image_3.tif]
