# Supplementary material for: IL-17 Producing Lymphocytes Cause Dry Eye and Corneal Disease With Aging in RXRα Mutant Mouse
Source: Front Med (Lausanne). 2022 Mar 23;9:849990. doi: 10.3389/fmed.2022.849990 (PMC8983848; doi:10.3389/fmed.2022.849990)
Supplement: Supplementary Table 1 — Antibodies in this study. [file Table_1.pdf]

Supplemental Table 1: Antibodies in this study

Antibody Type	Target	Clone	Host	Reaction	Conjugation	Company (Catalog No.)	Dilution
Primary	CXCL16	142417	Rat	Mouse	None	ThermoFisher (MA5-23869)	1:40
Primary	γδ T-Cell Receptor	GL3	Ham	Mouse	FITC	BD (553177)	1:30
Primary	RORγt	Q31-378	Mouse	Mouse	PE	BD (562607)	1:30
Primary	IL-17A	TC11-18H10	Rat	Mouse	PE	BD (559502)	1:30
Primary	Cyto-keratin 13 (KRT13)	CK-13	Rabbit	Mouse	None	ABclonal (A0411)	1:40
Primary	SPRR2	Polyclonal	Rabbit	Mouse	None	ABclonal (Custom-made using 'KCPEPCPPPKCPQPCPP' peptide sequence)	1:40
Secondary	IgG (H+L)	Polyclonal	Goat	Rat	Alexa Fluor 488	Jackson ImmunoResearch (112-546-143)	1:1000
Secondary	IgG (H+L)	Polyclonal	Goat	Rabbit	Alexa Fluor 488	Jackson ImmunoResearch (111-545-003)	1:1000
Secondary	IgG (H+L)	Polyclonal	Goat	Rabbit	Alexa Fluor 594	Jackson ImmunoResearch (111-585-003)	1:1000

Proteintech, Rosemont, IL; Thermofisher Scientific, Waltham, MA; Abcam, Waltham, MA

Abclonal, Woburn, MA; Becton Dickinson (BD), San Diego, CA; R&D Systems, Minneapolis, MN

Jackson ImmunoResearch, West Grove, PA; Ham = hamster
