# Supplementary material for: IL-17 Producing Lymphocytes Cause Dry Eye and Corneal Disease With Aging in RXRα Mutant Mouse
Source: Front Med (Lausanne). 2022 Mar 23;9:849990. doi: 10.3389/fmed.2022.849990 (PMC8983848; doi:10.3389/fmed.2022.849990)
Supplement: Supplementary Table 2 — Top differentially expressed genes in cell clusters. [file Table_2.pdf]

Supplemental Table 2. Top differentially expressed genes in cell clusters		
Cluster	Name	**Top 20 genes of each cluster**
0	MΦMHCII^{low}	Serpinb2, F5, Alox15, Basp1, Gadd45a, Ccl6, Cd24a, Rflnb, Ccl9, Tgm2, Irf2bp2, Cyp4f18, Vegfa, P2ry10, Arpc5, Plac8, Kctd12, Dnajb6, Thbs1, Itgam
1	Neutrophils-LCN2^{low}	Il1r2, Ifitm1, Tnfaip2, Nlrp3, Marcksl1, Csf3r, Ccrl2, Trem1, Clec4d, Clec4e, S100a8, Clec4n, S100a9, Il1b, Hdc, Resf1, Cd14, Acod1, C5ar1, Lrg1
2	γδ-T	Il17a, Trdc, Cxcr6, Areg, Cd163l1, Tcrg-C1, Icos, Cd3g, Rora, Emb<br><br>Cd3e, Trdv4, Blk, Ikzf3, Pdcd1, Il7r, Nrip1, Thy1, Cd3d, Tnfrsf9
3	Monocytes	Apoe, Lyz2, Mafb, Lgmn, Selenop, Ms4a6c, C1qa, F13a1, Chil3, Ctss, C1qb, Ctsc, Ifi207, Ifitm3, Ctsb, Mrc1, Ifi27l2a, Ccr2, Ly86, C1qc,
4	MΦ	Cd209a, Tnip3, Cd74, H2-Ab1, H2-Eb1, H2-Aa, Gm26917, Mgl2, Mt1, Cd83, Ifi30, Mt2, Etv3, Clec10a, H2-DMb1, H2-DMa, Syngr2, Sdc4, Ccnd1,<br><br>Crip1
5	ILC2	Il5, Gata3, Areg, Csf2, Rora, Lmo4, Ccl1, Nrip1, Furin, Itk, Il13, Il2ra,<br><br>Inpp4b, Tmem64, Calca, Ctla2a, Arg1, Hs3st1, Uhrf2, Il7r
6	NK cells	Gzma, Ccl5, AW112010, Nkg7, Irf8, Klra4, Il2rb, Prf1, Serpinb9, Klra8, Eomes, Gzmb, Ugcg, Klre1, Serpinb6b, Sh2d2a, Spry2, Ms4a4b, Txk, Klra9
7	CD4^{+} T cells	Cd28, Icos, Trac, Ets1, Rora, S1pr1, Ctla4, Thy1, Uhrf2, Il18r1, Trbc2, Ms4a4b, Ptpn22, Il7r, Trbc1, Emb, Tcf7, Lat, Ramp3, Cd3d
8	Neutrophils-LCN2^{high}	S100a9, S100a8, Lcn2, Stfa2l1, Wfdc21, Slfn4, Cstdc4, Lrg1, Hp, Clec4d, Slpi, Csf3r, Trem1, Acod1, Cxcr2, Hdc, Msrb1, Grina, Isg15,<br><br>Oasl2
9	cDC2-Retnla^{high}	Lpl, Retnla, Fn1, Lyz1, Fcrls, Ear2, Mt1, Pltp, Mt2, Lyz2, Cd74, Mgl2, H2-DMa, Crip1, H2-DMb1, H2-Ab1, Tnip3, Flrt3, Sdc3,<br><br>H2-Eb1

10	CD8$^+$ T cells	Ccl5, Xcl1, Il2rb, Ms4a4b, Gimap4, Trbc2, Ctsw, Serpinb6b, Klrc1, Nkg7, Gimap3, Ptpn22, Cd28, Ly6c2, AW112010, Klrk1, Ctla2a, Serpinb9, Txk, Ets1
11	B cells	Igkc, Cd79a, Ebf1, Ighm, Ighd, Scd1, Ms4a1, Cd79b, Mef2c, Ly6d, Pax5, H2-Ob, Satb1, Ccr7, Fcmr, Fcer2a, Ralgps2, Cd19, Cd55, H2-DMb2
12	cDC1	Cst3, Naaa, Wdfy4, Ppt1, H2-Ab1, H2-Eb1, 44442, Rab7b, H2-Aa, Plbd1, H2afz, Ifi205, Tmsb10, Cd74, Naga, Irf8, Mpeg1, Trim35, Eef1b2, Atpif1
13	Proliferating cell	Stmn1, Tubb5, Ighm, Top2a, Pclaf, Tuba1b, Mki67, Ube2c, H2afz, Hmgb2, H2afv, Birc5, Hist1h2ap, Anp32b, Dek, Tmsb10, Hist1h1b, Crip1, Nucks1, Hmgb1
14	Mast cells	Mcpt4, Cpa3, Cma1, Tpsb2, Mrgprb1, Jun, Serpinb1a, Kit, Egr1, Slc18a2, Gata2, Ccl2, Mrgprb2, Ube2v2, Cyp11a1, Tpsab1, Vwa5a, Fcer1a, Ccl7, Hs6st2
15	Treg cells	Ctla4, Tnfrsf4, Ikzf2, Il2ra, Icos, Hopx, Odc1, Cpm, Trac, Foxp3, Tnfrsf9, Rora, Cd2, Tnfrsf18, Cd28, Maf, Trp53inp1, Gata3, Ets1, Trbc2
16	Naive CD4+	Satb1, S1pr1, Dusp10, Lef1, Ccr7, Ms4a4b, Gimap3, Gramd3, Tcf7, Trbc2, Gimap6, Ablim1, Rpl12, Bcl2, Il7r, Ets1, Txk, Ms4a6b, Rps20, Trib2
17	Migratory DC (mDC)	Ccr7, Ccl22, Fscn1, Tbc1d4, Fabp5, Apol7c, Il4i1, Clic4, Cxcl16, Ccl17, Clu, Tmem123, Ifi30, Serpinb9, Tspan3, Nrp2, Socs2, Ccl5, Cacnb3, Serpinb6b
18	Plasmacytoid DC (pDC)	Siglech, Tcf4, Bst2, Ccr9, Ly6d, Klk1, Irf8, Ly6c2, Spib, Atp1b1, Lair1, Cox6a2, Lrp8, Ctsl, Cyb561a3, Bcl11a, Rpl31, Runx2, Grn, Mpeg1
